# Regulation of carotenoid and flavonoid biosynthetic pathways in *Lactuca sativa var capitate* L. in protected cultivation

**DOI:** 10.3389/fpls.2023.1124750

**Published:** 2023-02-14

**Authors:** Vanessa Harbart, Katja Frede, Maria Fitzner, Susanne Baldermann

**Affiliations:** ^1^ Department Plant Quality and Food Security, Leibniz Institute of Vegetable and Ornamental Crops (IGZ), Großbeeren, Germany; ^2^ Food Chemistry, Institute of Nutritional Science, University of Potsdam, Nuthetal, Germany; ^3^ Faculty of Life Sciences: Food, Nutrition and Health, Food Metabolome, University of Bayreuth, Kulmbach, Germany

**Keywords:** greenhouse, bioactive compounds, lettuce, flavonoid, carotenoid, metabolism, UV, crop cultivation

## Abstract

In the face of a growing world population and limited land, there is an urgent demand for higher productivity of food crops, and cultivation systems must be adapted to future needs. Sustainable crop production should aim for not only high yields, but also high nutritional values. In particular, the consumption of bioactive compounds such as carotenoids and flavonoids is associated with a reduced incidence of non-transmissible diseases. Modulating environmental conditions by improving cultivation systems can lead to the adaption of plant metabolisms and the accumulation of bioactive compounds. The present study investigates the regulation of carotenoid and flavonoid metabolisms in lettuce (*Lactuca sativa var capitate* L.) grown in a protected environment (polytunnels) compared to plants grown without polytunnels. Carotenoid, flavonoid and phytohormone (ABA) contents were determined using HPLC-MS and transcript levels of key metabolic genes were analyzed by RT-*q*PCR. In this study, we observed inverse contents of flavonoids and carotenoids in lettuce grown without or under polytunnels. Flavonoid contents on a total and individual level were significantly lower, while total carotenoid content was higher in lettuce plants grown under polytunnels compared to without. However, the adaptation was specific to the level of individual carotenoids. For instance, the accumulation of the main carotenoids lutein and neoxanthin was induced while the β-carotene content remained unchanged. In addition, our findings suggest that the flavonoid content of lettuce depends on transcript levels of the key biosynthetic enzyme, which is modulated by UV light. A regulatory influence can be assumed based on the relation between the concentration of the phytohormone ABA and the flavonoid content in lettuce. In contrast, the carotenoid content is not reflected in transcript levels of the key enzyme of either the biosynthetic or the degradation pathway. Nevertheless, the carotenoid metabolic flux determined using norflurazon was higher in lettuce grown under polytunnels, suggesting posttranscriptional regulation of carotenoid accumulation, which should be an integral part of future studies. Therefore, a balance needs to be found between the individual environmental factors, including light and temperature, in order to optimize the carotenoid or flavonoid contents and to obtain nutritionally highly valuable crops in protected cultivation.

## 1 Introduction

Even small environmental changes can alter a plant’s metabolome; in this respect, plant metabolism is still a black box. To improve cultivation systems and nutritional quality of horticultural crops, it is crucial to understand the regulation of a plant’s metabolism. By 2050, the human population is predicted to reach 9.75 billion (Prb, 2022), therefore, efficient production of horticultural crops is important to ensure food security. Moreover, not only crop yields should be addressed, but also new approaches to producing nutrient-rich crops.

Protected cultivation systems, which is the term used for growing crops such as vegetables, in greenhouses, polytunnels, or row covers, can be one approach to increasing yields as well as improving the nutritional quality of crops. In 2019, an area of 5,630,000 ha of land was used for protected agriculture worldwide (World Greenhouse Vegetable Statistics, 2019), while in Europe (2020), 1,140,913 ha of agricultural land was under protective covers compared to 288,051,555 ha of cropland [Food and Agriculture Organization Corporate Statistical Database (FAOSTAT) (2020)]. This represents an increase of around 5% in agricultural land under protective covers in six years (since 2014) [Food and Agriculture Organization Corporate Statistical Database (FAOSTAT) (2020)]. Certainly, greenhouse area is constantly increasing for vegetable production, and certain greenhouse cultivars achieve much higher yields (e.g. tomato cultivars with 40% higher yields compared to old-cultivars). Thus, much higher vegetable greenhouse production is estimated in the future (Marcelis and Heuvelink, 2019). Protected cultivation of crops improves yields and enables higher productivity due to extending seasonal production times compared to open fields (Gruda, 2005). Furthermore, such crop production offers opportunities for sustainable cultivation, which is fundamental for future food production. For example, strategic location of greenhouses with short transportation distances to reduce food miles or in areas unsuitable for open-field cultivation could be considered (Zhou et al., 2021), as well as resource-efficient water use, particularly in hotter climates (Irusta et al., 2009).

Another important aspect of sustainable crop production is the choice of covering material. Different materials generate different radiometric and physical properties and thus individual selection that depends on regions, seasons, or crop species may be most beneficial (Maraveas, 2019). For polymer-based greenhouse covers, the incorporation of additives offers additional possibilities for desired properties. For example, UV blockers are added to polymers to reduce the transmission of UV light, which can cause plant damage due to pests and diseases (Katsoulas et al., 2020), whereas antifogging additives can improve light transmission and avoid microbiological contamination due to the prevention of water droplets on the plant facing side (Irusta et al., 2009). The use of protected cultivation also enables control of temperature and light regimes, in particular, to trigger adaptations in the metabolic response of plants. Thus, the selection of different greenhouse covers, including incorporating property-improving additives, can provide another often-neglected possibility for improving the nutritional quality of horticultural crops (Ahmadi et al., 2018; Petropoulos et al., 2019; Harbart et al., 2022). Therefore, understanding the metabolic responses and regulation of bioactive compounds is crucial for developing and selecting suitable materials.

Bioactive compounds such as plant secondary metabolites are ubiquitously distributed in plants. Although not essential for human health, they are associated with several beneficial properties when included in human nutrition. For example, epidemiological studies have revealed that both carotenoids and flavonoids have beneficial effects on non-transmissible diseases such as cardiovascular diseases or cancer (Hertog et al., 1993; Kim and Je, 2017; Milani et al., 2017; Rees et al., 2018). In addition, certain carotenoids are precursors of vitamin A, and the xanthophylls lutein and zeaxanthin have been shown to affect the development and progression of age-related macular degeneration (Eisenhauer et al., 2017). *In planta*, both bioactive compounds have protective functions against photoinhibition, and carotenoids in particular are involved in photosynthesis (Agati and Tattini, 2010; Cazzonelli, 2011).

**Figure 1:**
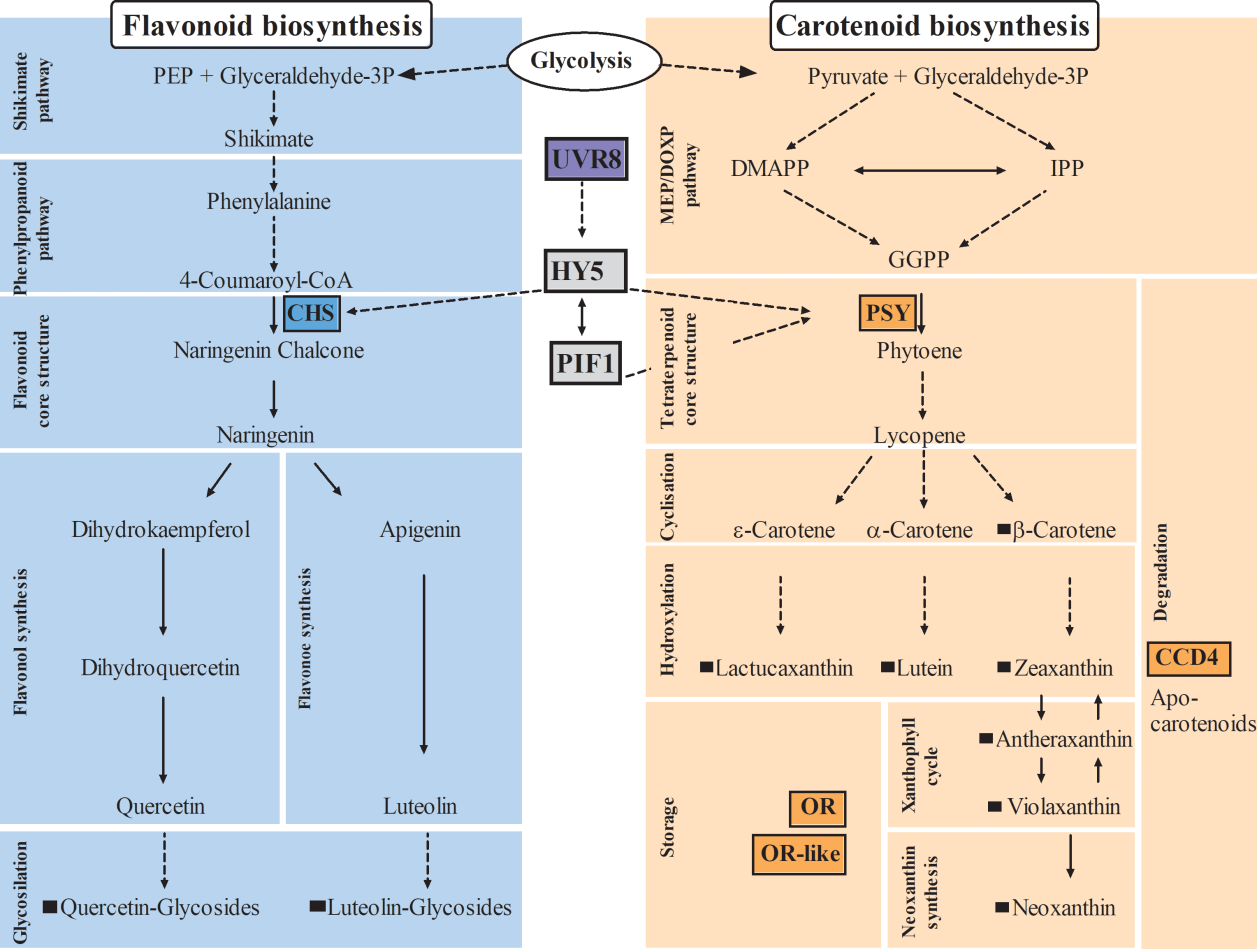
Impact on carotenoid and flavonoid biosynthetic pathways in plants. Arrows with dashed lines indicate more than one reaction, arrows with continuous lines indicate one reaction; key enzymes of flavonoid and carotenoid pathways are highlighted by colored boxes; black squares indicate metabolites identified in lettuce in this study. CHS, Chalcone synthase; PSY, Phytoene synthase; CCD4, Carotenoid cleavage dioxygenase4; OR, Orange protein; UVR8, Ultraviolet resistance locus8; HY5, Elongated hypocotyl5; PIF1, Phytochrome interacting factor1; DMAPP, dimethylallyl diphosphate; IPP, isopentenyl diphosphate; GGPP, geranylgeranyl diphosphate; MEP/DOXP, mevalonate/non-mevalonate pathway.

The biosynthesis of flavonoids follows the shikimate pathway, while carotenoids are synthesized through mevalonate and non-mevalonate pathways (KEGG pathway database, **Figure 1**), both biosynthetic pathways are well studied and highly conserved in the plant kingdom. However, it is less well understood how plants regulate the biosynthesis, accumulation, and degradation of these compounds. In addition, many studies have been conducted in model organisms such as *Arabidopsis thaliana*; however, many mechanisms, particularly in carotenoid pathway, seem to be species- and tissue-specific (Ohashi-Kaneko et al., 2007; Shumskaya et al., 2012; Lätari et al., 2015). Consequently, there is a need for studies in horticultural crops in order to effectively transfer knowledge from plant model systems.

Light and temperature were identified as the most important factors controlled by greenhouses (Fadel et al., 2016). Frequently, solar radiation is the only source of light in low-tech protected cultivation, such as in simple polytunnels. Plants respond to light using different pathways. For example, plants detect blue, red, or UV light through various photoreceptors that trigger transcriptional cascades for metabolite adaptation such as carotenoid and flavonoid levels (Rizzini et al., 2011; Morales et al., 2013; Toledo-Ortiz et al., 2014; Yang et al., 2018; Stanley and Yuan, 2019; Tissot and Ulm, 2020). In addition, light modulating processes such as shade avoidance are also part of the physiological response (Bou-Torrent et al., 2015). Plants need to adjust their metabolites to a given environment and direct their metabolic flux and energy input towards synthesizing the most favorable compounds. Thus the regulation of bioactive compounds such as carotenoids and flavonoids, which share some functions in the plant such as protection or attraction (anthocyanins), is probably interrelated (Cao et al., 2015). Understanding such crosstalk between pathways can help improve the nutritional quality of horticultural crops and such knowledge can contribute to targeted decision making about cultivation conditions.

The aim of this study was to advance our understanding of the metabolic regulation of carotenoids and flavonoids as nutritionally valuable bioactive compounds in lettuce. We grew lettuce without or under polytunnels, testing two different covering materials, with and without incorporated antifogging additives. Climatic conditions were monitored during the experiments, and carotenoid and flavonoid profiles as well as the phytohormone (ABA) content were determined by high performance liquid chromatography coupled with mass spectrometry (HPLC-MS). Promising target gene candidates encoding key biosynthetic enzymes of both metabolic pathways as well as light-depended transcription factors were analyzed by RT-*q*PCR. We demonstrate that different mechanisms, at both the transcriptional (flavonoids) and posttranscriptional (carotenoids) levels, influence the accumulation of these bioactive compounds in lettuce cultivated under polytunnels. The inverse relationship between flavonoid and carotenoid levels is probably caused by physicochemical mechanisms rather than a shared transcriptional signaling pathway.

## 2 Material and methods

### 2.1 Plant growth and cultivation

Lettuce (*Lactuca sativa var capitata* L., cultivar ‘Veronique’) seeds were obtained from Samenhaus Müller GmbH (Wildeck-Bosserode, Germany) and were sown in trays with soil (substrate type P, pH 5.9, N 120 mg L^-1^, PO_4_^2-^ 120 mg L^-1^, K 170 mg L^-1^, Mg 120 mg L^-1^, Einheitserde classic, Einheitserde Werkverband e.V., Sinntal-Altengronau, Germany). After reaching two-leaf-stage, seedlings were transplanted into pots (diameter 13 cm) filled with soil (substrate typ T, pH 5.9, N 183 mg L^-1^, P_2_O_5_ 135 mg L^-1^, K_2_O 212 mg L^-1^, salinity 1.23 g L^- 1^, Einheitserde classic, Einheitserde Werkverband e.V., Sinntal-Altengronau, Germany). The pots were then placed in a cabin in a glasshouse located at Leibniz Institute of Vegetable and Ornamental Crops (Grossbeeren, 52°20’5N 13°18’35.3”E). Three experimental repetitions were performed in April, May and September 2021. The glasshouse cabin temperature and the relative humidity was set to 22°C and 70%, controlled by open vents. No supplemental artificial light was used.

Polytunnels, placed in the glasshouse, were covered using three-layered polyethylene films (low-density polyethylene/linear low-density polyethylene/14% ethylene butyl acrylate (middle layer), 180 μm thickness, CONSTAB polyolefin additives GmbH, Rüthen, Germany), four films contained antifogging additives (Sabostat A 300 and Atmer 103, 0.35%) whereas the other four were additive free (**Figure S1**). Thus, in total, each experiment was performed with four biological replicates per cultivation condition (polytunnel with antifog, antifog-free polytunnel and without polytunnel). Five lettuce plants were cultivated under each polytunnel as well as 20 lettuce plants without. To avoid an influence due to position, the lettuce plants were randomized twice a week and the positions of the polytunnels were randomized once halfway through the experimental period. Harvesting was carried out about 20 days after transplanting. The five largest leaves per plant were cut off, the midrib was removed, and a sample containing leaves from four lettuce plants was immediately frozen in liquid nitrogen. Two samples were taken per biological replicate, since one was used for metabolite analysis and the other for RNA extraction. Samples for metabolite analysis were lyophilized and stored vacuum-packed at ambient temperature in the dark until further analysis. Homogenization was performed using a mill (Retsch^®^ MM 400, 45 s, 2 repetitions at 25 1 s^-1^). The fresh material was homogenized for RNA extraction using a mortar and pistil under liquid nitrogen and stored at -80°C.

### 2.2 Monitoring of climatic conditions

The temperature, relative humidity and the PPFD (photosynthetic photon flux density) were monitored during the experiments in the greenhouse cabin as well as under the polytunnels in the greenhouse. To detect temperature and relative humidity, sensors (PT - 100 type B sensor, Galltec Mess- und Regeltechnik GmbH, Bondorf, Germany, MELA Sensortechnik GmbH Mohlsdorf-Teichwolframsdorf, Germany). were placed under four polytunnels Monitoring in the greenhouse cabin was performed by an aspiration psychrometer (Type ELAU KlimaExpert, KE-PTFF-8024-OF, Elektro- und Automatisierungsanlagen Pierre Ambrozy, Gatersleben, Germany). Five PAR sensors (photosynthetic active radiation, LI-190R Quantum Sensor, LI-COR Biosciences GmbH, Germany) were used, four placed under the polytunnels and one in the greenhouse cabin. Furthermore, the UVA and UVB transmittances inside the polytunnels were determined once during the experiment using a spectrometer (Optic Spectrometer, Ocean Optics Inc., Ostfildern, Germany), UV/VIS transmission spectra of both films were also determined before use (**Figure S2**) using photospectrometer (Lambda 365, PerkinElmer, Inc., Waltham, USA).

### 2.3 Chemicals and standards

Ethanol (≥ 99.9%, LiChrosolv^®^), tetrahydrofuran (THF, ≥ 99.9%, LiChroSolv^®^), n-hexane (SupraSolv^®^), dichloromethane (< 99.8%, SupraSolv^®^), chlorophyll a and b (analytical standards) and norflurazone (Pestanal^®^, analytical standard) were obtained from Merck KGaA (Darmstadt, Germany). *Tert*-butyl methyl ether (≥ 99.9%, Rotisolv^®^), 2-propanol (≥ 99.9%, Rotisolv^®^), ammonium acetate (≥ 98%) and acetic acid (100%, Supra Quality) were purchased from Carl Roth GmbH (Karlsruhe, Germany). Methanol (Chemsolute^®^) and acetonitrile (Chemsolute^®^) were purchased from Th. Geyer GmbH & Co. KG (Renningen, Germany). Carotenoid standards were obtained from CaroteNature GmbH (Münsingen, Switzerland) and flavonol glycosides from PhytoLab GmbH & Co. KG (Vestenbergsgreuth, Germany). Abscisic acid (ABA) standard was purchased from Sigma Aldrich Chemie GmbH (Taufkirchen, Germany) and (+)-abscisic acid-d6, ≥ 98%) from Toronto Research Chemicals (North York, Canada). All solvents were of LC-MS quality and the water was of ultra-pure quality.

### 2.4 Analysis of carotenoid metabolic flux with norflurazon treatment

Lettuce was grown in September and October 2021 in two independent experiments under the same conditions as mentioned above. Norflurazon treatment was performed at the lettuce 8-leaf-stage after 10 days of polytunnel cultivation, as described previously for leaves of *Arabidopsis thaliana* (Koschmieder and Welsch, 2020). In detail, two leaves per plant were transferred in an aqueous norflurazon solution (70 µM in aqueous 0.125% 2-propanol) and two leaves in water (aqueous 0.125% 2-propanol) as controls. The leaves were incubated in darkness for 2 h. Afterwards, the norflurazon solution was changed to 10 µM and the leaves were incubated for another 4 h in daylight (PPFD: approximately 140 µmol s^-1^ m^-2^) under each cultivation condition. Lettuce leaves from a total of five plants were collected as one sample (pool sample per polytunnel or without polytunnel), the midrib was removed and samples were frozen in liquid nitrogen and stored at -60°C until further analysis. Sample preparation was performed as described above.

### 2.5 Analysis of flavonoid glycosides *via* HPLC-DAD-MS/MS

10 mg of homogenized sample was extracted three times with methanol/water (3:2, v/v) as previously described (Neugart et al., 2019). Combined supernatants were subsequently dried using a Speedvac (SPD111V, Thermo Scientific). The dried samples were redissolved in 200 µL methanol/water (1:9, v/v) and filtered through Spin-X cellulose acetate filters (0.22 µM) tubes. The extracts were analyzed by Agilent 1260 Infinity II HPLC equipped with an Ascentis^®^ Express F5 column (150 mm × 4.6 mm, 5 μm, Supelco, Sigma Aldrich Chemical Co., St Louis, MO, USA). The flavonoid glycosides were detected using a photodiode array detector at wavelength 370 nm. Compounds were eluted using solvent A: 0.5% acetic acid and solvent B: acetonitrile in gradient mode. A Bruker amazon SL ion trap mass spectrometer was used to determine mass spectra and perform fragmentation of the separated compounds. Ionization was performed by ESI (electrospray ionization) in negative polarity. The flavonoid glycosides were identified based on their absorption maxima, mass spectra and fragmentation pattern either comparing with authentic standards and with literature data (**Table S1**). Quantification was performed using external calibration at 370 nm. Quercetin derivatives were quantified using quercetin-3-glucoside and luteolin derivatives as luteolin-7-glucoside.

### 2.6 Analysis of carotenoids *via* HPLC-DAD-ToF-MS

For carotenoid analysis, 5 mg of homogenized samples were weighed out, followed by three times extraction with 500 µL tetrahydrofuran/methanol (1:1, v/v) as previously described (Harbart et al., 2022). The collected and combined supernatants were dried under a nitrogen stream and redissolved in 250 µL dichloromethan/2-propanol (1:5, v/v). After filtration through PTFE filters (0.2 µm) the extracts were analyzed by HPLC-DAD-ToF-MS using an Agilent Technologies 1290 Infinity UHPLC coupled with an Agilent Technologies 6230 ToF LC/MS. Briefly, the separation was performed in gradient mode on a C30 column (YMC Co. Ltd, Kyoto, Japan, YMC C30, 100 × 2.1 mm, 3 μm) with eluents containing A: methanol/water (96:4, v/v) and B: methanol/*tert*-butyl methyl ether/water (6:90:4, v/v/v) both added with ammonium acetate (20 mM) to enhance the ionization. Ionization was achieved using a multimode ion source in positive polarity. Carotenoids were (tentatively) identified based on their specific absorption and mass spectra, in comparison with the literature or authentic standards (**Table S2**). The quantification of carotenoids was calculated *via* external calibration with authentic standards at wavelength 450 nm.

### 2.7 Analysis of phytoene *via* HPLC-QToF-MS

Phytoene was extracted from 5 mg homogenized samples by the modified method of Frede and Baldermann (2022). At first, 200 µL ethanol and 100 µL water were added followed by 1 min sonication. Phytoene was then extracted twice with 500 µL and 300 µL hexane. The collected supernatants were evaporated to dryness under a nitrogen stream. Finally, samples were redissolved in 375 µL dichloromethane/2-propanol (1:5, v/v) and filtered through PTFE filters (0.2 µm). The instrument settings were applied in general as for carotenoids, but with the following modifications. A 1290 Infinity HPLC-DAD coupled with a 6546 QToF-MS (Agilent Technologies, Waldbronn, Germany) was used for the measurements. In contrast to the other carotenoids, phytoene was detected using a QToF system equipped with an APCI source (atmospheric pressure chemical ionization). Ions were detected in positive polarity with a gas temperature of 325°C, vaporized at 350°C, drying gas with a flow of 8 L min^-1^ and nebulizer at 35 psi. Corona voltage was set to 3500 V and a current of 6.5 µA. Phytoene was identified comparing mass spectra with an authentic standard (**Table S2**). Quantification was performed *via* external calibration with the authentic standard using the extracted masse of the *m/z* = 545.5081 [M+H]^+^ ion. Phytoene was quantified as the sum of the isomers present.

### 2.8 Analysis of abscisic acid *via* HPLC-MS/MS

Abscisic acid (ABA) extraction was performed according to the method by Errard et al. (2016) with modifications. ABA was extracted from 10 mg homogenized sample using methanol/water (3:2, v/v). Deuterated ABA was added as an internal standard followed by sonication in cold. Combined supernatants were filtered through PTFE filters (0.2 µm) and diluted with 0.1% acetic acid in ultrapure water (1:1, v/v). The extracts were analyzed using an Agilent Technologies 1260 Infinity HPLC coupled with a triple quadrupole, Q-Trap^®^ 6500-MS/MS system (AB Sciex LLC, Framingham, USA) equipped with a Zorbax Eclipse Plus C18 column (1.8 µm, 2.1 mm x 50 mm; Agilent Technologies, Waldbronn, Germany). Elution was performed in gradient mode using solvent A: 0.1% acetic acid and solvent B: acetonitrile and 0.1% water. Ionization was performed in negative mode using ESI (electrospray ionization) at 500°C with the following settings: ionization voltage, -4,500 V; curtain gas, 50 psi; drying gas, 50 psi; nebulizer gas, 50 psi; auxiliary gas, 65 psi; and multi reaction monitoring (MRM) at a dwell time of 0.3781 s. Identification and quantification were based on MRM transitions (263→153 quantifier, 263→203 and 263→122 qualifier). ABA was quantified using external calibration with internal standard.

### 2.9 Gene expression analysis *via* RT-*q*PCR

Approximately 50 mg of powdered fresh tissue was weighed out for RNA extraction using the RNeasy Plus Mini Kit (Qiagen, Hilden, Germany) according to manufacturer’s instructions, with an on-column DNAse I digestion. RNA concentration was determined spectrophotometrically with Nanodrop at 260 nm (ND1000, Thermo Fisher Scientific, Waltham, MA, USA) with a desired ratio of 260/280 ∼ 2.0 and 260/230 ∼ 2.0-2.3. Additionally, the quality of selected RNA samples was checked using the bioanalyzer (2100 bioanalyzer, Agilent Technologies). A RIN value of ≥ 7.3 was accepted for further usage. The cDNA was synthesized with the SuperScript III reverse transcriptase (Thermo Fisher Scientific, Waltham, MA, USA) and oligo (dT)12−18 primers as described by the manufacturer using 250 ng total RNA. Primers for target and reference genes were designed using sequences available at Phytozome or NCBI (National Center for Biotechnology Information; **Table S4**). The primer amplification efficiencies were determined with cDNA dilution analysis. Detailed information about primer sequences and efficiencies can be found in **Table S3**. The stability of selected reference genes (actin 7, ubiquitin-conjugating enzyme E2 A and elongation factor 1-alpha) was checked (M value <0.5; coefficient variance <0.25). The RT-*q*PCR experiments were performed in triplicates using 3 μL diluted cDNA (1:10), 5 μL 2× SensiMix SYBR Low-ROX (Bioline, Luckenwalde, Germany) and 2 μL of 2 μM primer. Experiments were conducted with a CFX96 Real-Time PCR Detection System (Bio-Rad Laboratories, Inc., Hercules, CA, USA) with the following thermal cycling conditions: 95°C for 10 min, 39 cycles of 95°C for 15 s, 58°C for 15 s followed by 72°C for 30 s and a subsequent melting curve analysis. For the analysis of *CCD4* and *OR* family genes, an adjusted annealing temperature of 60°C was used. Data were evaluated using the ΔΔCq method according to Vandesompele et al. (2002); Pfaffl (2004) with the geometric mean of the three reference genes. The expression of genes of interest were calculated as *n*-fold changes relative to gene expression in the lettuce samples grown without polytunnels.

### 2.10 Statistical analysis

The SigmaPlot 14.0 software (Systat, Erkrath, Germany) was applied for statistical analysis. Data were compared by one-way ANOVA followed by Tukey HSD *post hoc* test assuming normal distribution and variance homogeneity. If the assumption did not apply, a Kruskal-Wallis one way ANOVA on ranks was performed. Significant differences were considered at p ≤ 0.05 and are indicated by different letters or asterisk. Outlier identification was performed by a Grubbs test, assuming outliers with a G ≥ 1.4925 for a representative sample size of n = 4. Data are presented as mean ± standard error unless otherwise stated.

## 3 Results

### 3.1 Changing climate conditions caused by protected cultivation

To assess the different light transmittance, reflectance, scattering and heat absorption properties of the materials used in protected cultivation, climatic conditions were evaluated for each experimental setup. We found that regardless of the experimental repetition (in April, May or September), similar differences when comparing cultivation with and without polytunnels were observed for all factors examined (**Table 1**). In detail, a 1.31-fold lower daily light integral (DLI), 1.1-fold higher temperature, and 1.89-fold higher relative humidity were determined under polytunnels (with and without antifogging additives). However, absolute values varied between experimental repetitions due to seasonal changes. Interestingly, temperature in general was not significantly different between repetitions, but DLI, photoperiod, and relative humidity were affected, with higher DLI and photoperiod and lower relative humidity in May and September compared to April. Although no temperature differences due to seasonal changes were determined for lettuce without polytunnels, significant differences in temperature were observed due to the use of polytunnels. Notably, the temperature differences under polytunnels were consistent with changes in DLI. Hence, there is a close relationship between temperature and DLI, particularly under the polytunnels, which is a phenomenon known as the greenhouse effect (Baudoin et al., 2013).

**Table 1:** Lettuce cultivation characteristics of three independent experimental repetitions in April (1), May (2) and September (3).

Lettuce cultivation characteristics	Experimental repetition	Lettuce cultivation condition		
		Without polytunnel	Polytunnel with antifog	Polytunnel without antifog
Daily light integral (mol m^-2^ d^-1^)	1	7.23 ± 1.91^AB,a^	5.81 ± 1.63^AB,b^	5.33 ± 1.41^B,b^
	2	9.01 ± 3.34^A,a^	7.10 ± 2.36^A,b^	7.07 ± 2.22^A,b^
	3	5.97 ± 3.41^B,a^	4.30 ± 0.31^B,ab^	2.10 ± 0.21^C,b^
Photoperiod (daylight, h)	1	13.37 ± 0.41^B^		
	2	15.14 ± 0.55^A^		
	3	12.67 ± 0.60^C^		
Temperature (°C)	1	22.61 ± 0.54^a^	25.29 ± 1.61^AB,b^	25.19 ± 1.43^AB,b^
	2	23.04 ± 1.17^a^	26.06 ± 2.26^A,b^	26.28 ± 2.38^A,b^
	3	22.57 ± 0.80^a^	24.07 ± 0.78^B,b^	24.05 ± 0.77^B,b^
Relative humidity (%)	1	42.98 ± 3.90^C,a^	86.30 ± 8.77^B,b^	91.79 ± 8.89^c^
	2	46.67 ± 5.76^B,a^	90.43 ± 5.75^AB,b^	92.13 ± 5.14^b^
	3	56.52 ± 5.67^A,a^	95.48 ± 1.82^A,b^	95.48 ± 1.67^b^

Lettuce was grown without or under polytunnels with and without antifogging additives. Data are shown as averaged values (mean ± SD) monitored continuously over the experimental period. Capital letters indicate significant differences (p ≤ 0.05) between the three repetitions within similar cultivation conditions. Lower case letters indicate significant differences (p ≤ 0.05) between cultivation conditions within one experimental repetition; no letters indicate absence of significance.

**Figure 2:**
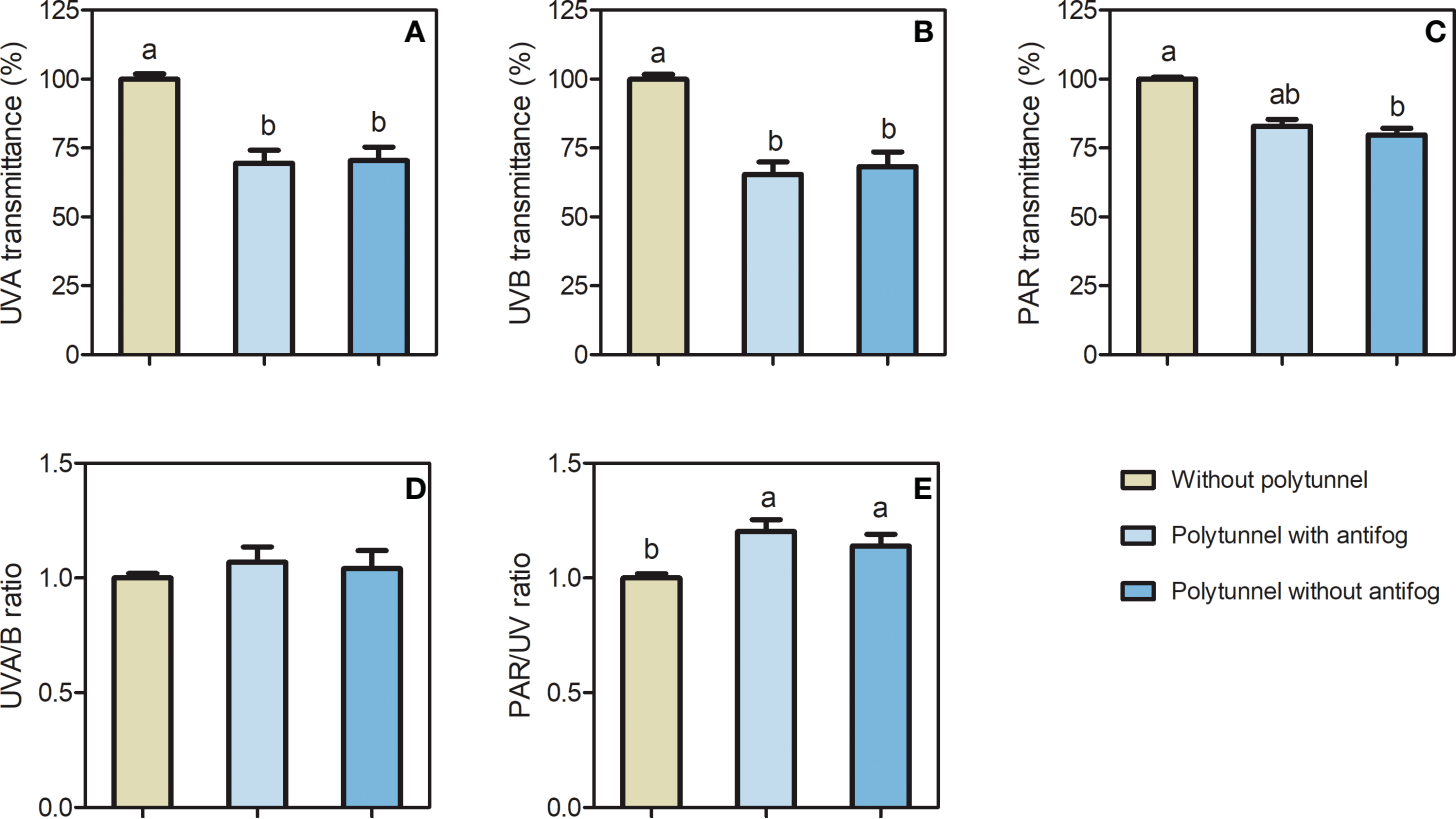
Differences in light intensity and light quality without and under polytunnels. **(A, B)** UV, and **(C)** PAR transmittance (%) of polytunnel materials with and without antifogging additives compared to without polytunnels, **(D)** UVA/B ratio, and **(E)** UV/PAR ratio. Measurements were conducted within the second experimental repetition (in May). Data represent mean ± SD (n = 4). Different letters indicate significant differences (p ≤ 0.05); no letters indicate no significance. UV, ultraviolet; PAR, photosynthetic active radiation.

The material of the polytunnels reduced certain light transmission (**Figure S2**), which led to differences in light intensity and light quality among the polytunnels, compared to cultivation without polytunnels (**Figure 2**). A 1.43-fold lower UVA and 1.50-fold lower UVB transmittance were determined due to either covering material, while light intensity (PAR) was 1.25-fold lower when comparing cultivation without polytunnels and polytunnels without antifog. Interestingly, no difference in PAR light intensity was observed for cultivation without polytunnels and polytunnels with antifog. Additionally, the use of polytunnels did not affect UVA to UVB ratios as well as far-red to red light ratio (**Figure S3**), but the PAR to UV ratio was 1.17-fold higher under polytunnels.

We assume that light is the most crucial factor here likely to affect the metabolic processes in the protected cultivation of lettuce, firstly, due to the strong direct correlation between higher DLI and increasing temperature under the polytunnels compared to without (**Figure S7**), and secondly, due to altered light quality as a consequence of the transmission properties of the films. As a result, we decided to present the data obtained from experimental repetition 1, since there were no significant differences in either DLI or photoperiod in experimental repetitions 2 and 3.

Since light and temperature are key factors that differ through protected cultivation, these require consideration for evaluating the regulation of metabolic pathways of bioactive compounds in horticultural crops. Notably, the two factors are interrelated when using covers and it is difficult to evaluate them separately.

### 3.2 Polytunnel cultivation affects the flavonoid glycoside content

**Figure 3:**
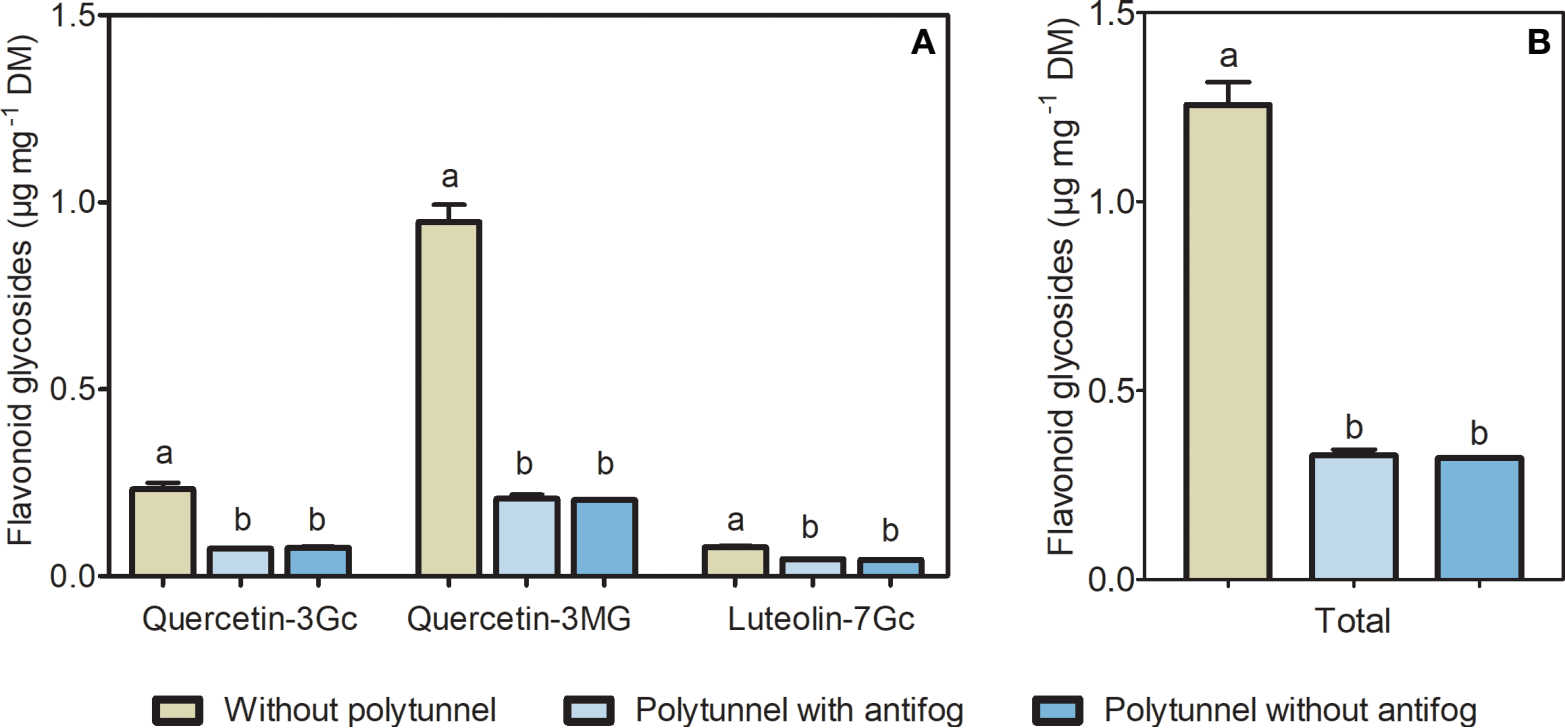
Flavonoids in lettuce cultivated without or under polytunnels. **(A)** Individual, and **(B)** total content of flavonoid glycosides (μg mg^-1^ DM) in lettuce grown without or under polytunnels with and without antifogging additives. The first experimental repetition in April is shown. The data are expressed as mean ± SE (n = 4). Significant differences (p ≤ 0.05) between treatment of individual compounds and total content are indicated by different letters. Gc, glucuronide; MG, malonyl glucoside.

Light intensity, quality, and temperature, among other environmental factors, can modulate the flavonoid content in horticultural crops. To unravel changes in flavonoids caused by altering climate conditions in protected cultivation, we analyzed flavonoid glycosides in lettuce cultivated under polytunnels (with and without antifog) or without. Two flavonols (quercetin derivatives) and a flavone (luteolin derivative) were identified in lettuce as glycosylated and acylated with sugar and organic acid moieties. Quercetin was present as glucuronide and malonyl-glucoside and luteolin was detected as a glucuronide derivative (**Table S1** and **Figure S6**). The cultivation of lettuce under polytunnels resulted in 3.87-fold lower amounts of total flavonoids, as well as reduced amounts of individual compounds (quercetin glucuronide 3.10-fold, quercetin malonyl-glucoside 4.61-fold, luteolin glucuronide 1.72-fold; **Figure 3**). This was not dependent on the antifogging additives. Furthermore, lower flavonoid content in lettuce grown under polytunnels compared to without were measured in all experimental repetitions (**Figure S8**).

### 3.3 Polytunnel cultivation affects the carotenoid content

The bioactive carotenoids act as photosynthetic pigments, and, in particular, are responsive to differences in the light regime and temperature as well as other environmental factors. Polytunnel cultivation affected carotenoid content of lettuce significantly. Major carotenoids such as β-carotene and lutein were (tentatively) identified in lettuce, besides phytoene, violaxanthin, and neoxanthin and other minor carotenoids (**Table S2**, **Figures S4** and **S5**). In addition, the lettuce-specific carotenoid lactucaxanthin was (tentatively) identified. Phytoene, violaxanthin and neoxanthin contents are represented as the sum of their detected isomers. Besides carotenoids as photosynthetic pigments, chlorophyll a and b were also detected (**Figures S13**-**15**).

**Figure 4:**
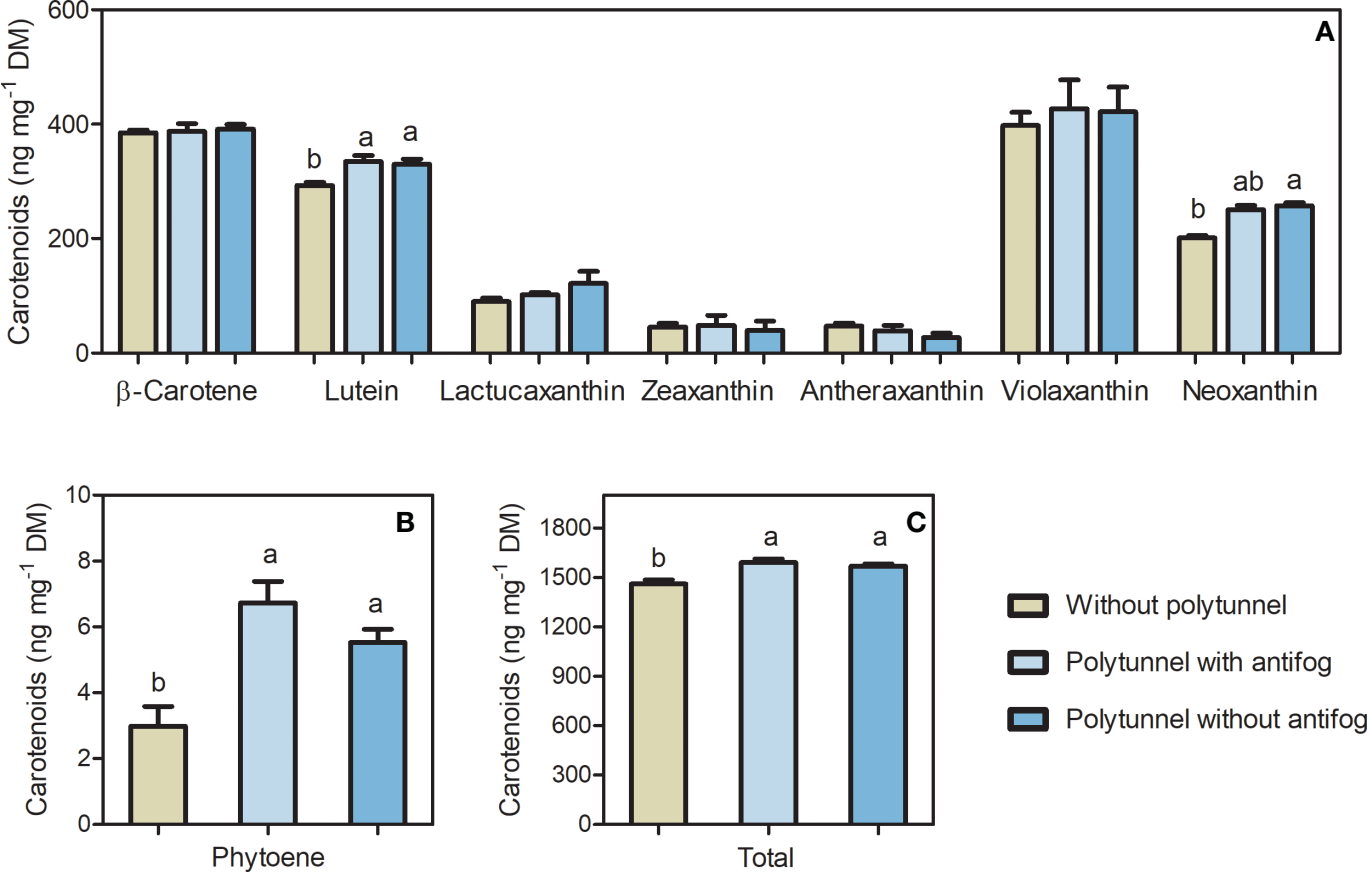
Total carotenoid content affected by polytunnel cultivation. Carotenoid content (ng mg^-1^ DM) in lettuce grown without or under polytunnels with and without antifogging additives. **(A)** Individual carotenoids β-/ϵ-branch and downstream, **(B)** upper pathway metabolite phytoene, and **(C)** total carotenoids. First experimental repetition in April is shown. The data are expressed as mean ± SE (n = 4). Significant differences (p ≤ 0.05) between treatment of individual compounds and total content are indicated by different letters; no letters indicate absence of significance.

Total carotenoid content was affected due to polytunnel cultivation (**Figure 4**). In particular, lettuces grown under polytunnels showed 1.08-fold higher total carotenoid content compared to growth without polytunnels. At the individual level, lutein (1.13-fold) and phytoene (2.05-fold) content were higher with polytunnel cultivation than without. However, neoxanthin content was 1.55-fold higher in lettuces grown under antifog-free polytunnels than without polytunnels. Albeit not significant, the xanthophylls lactucaxanthin and violaxanthin showed similar tendencies in polytunnel grown lettuces. Trends of higher carotenoid levels were also evident in each experimental repetition (1 to 3), however except for lutein, changes at the level of the individual carotenoids also occur between the repetitions (**Figure S9**).

In summary, the use of polytunnels for lettuce cultivation resulted in lower overall flavonoid glycoside content and higher contents of carotenoids, although differences occur at the level of individual carotenoid compounds.

### 3.4 RNA transcript levels of carotenoid and flavonoid pathway genes and transcription factors

**Figure 5:**
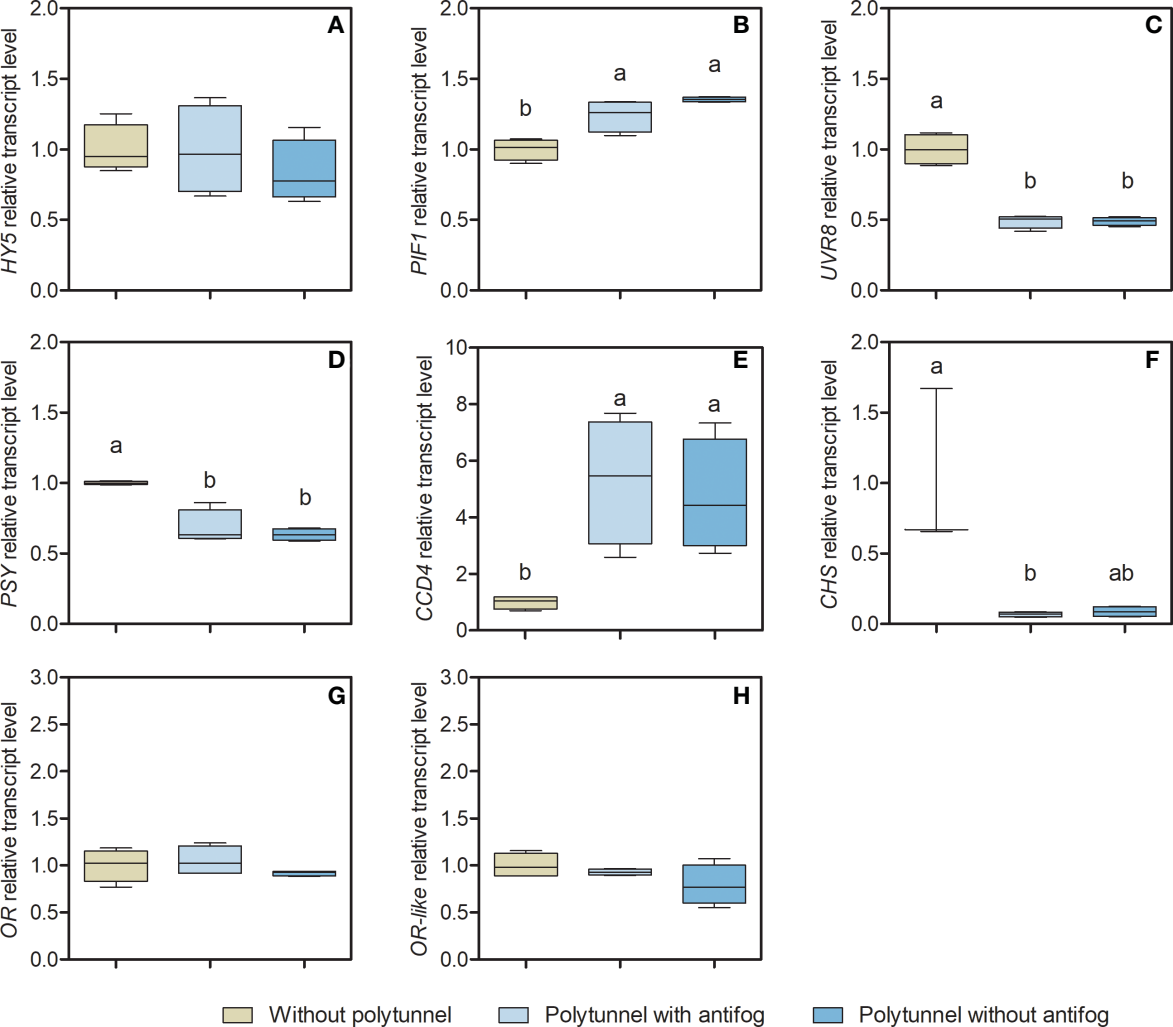
Gene transcripts for key enzymes of the core carotenoid and flavonoid biosynthesis pathways. Transcript levels of **(A)** *HY5*, **(B)** *PIF1*, **(C)** *UVR8*, **(D)** *PSY*, **(E)** *CCD4*, **(F)** *CHS*, **(G)** *OR*, and **(H)** *OR-like* in lettuce grown without or under polytunnels with and without antifogging additives. The first experimental repetition in April is shown. The data are expressed as Box-Whisker-Plots (n = 4); Whiskers show maximal and minimal values. Data was normalized to lettuce grown without polytunnels. Different letters indicate significant differences (p ≤ 0.05) of transcripts under different cultivation conditions; no letters indicate absence of significance. *HY5*, Elongated hypocotyl5; *PIF1*, Phytochrome interacting factor1; *UVR8*, Ultraviolet resistance locus8; *PSY*, Phytoene synthase; *CCD4*, Carotenoid cleavage dioxygenase4; *CHS*, Chalcone synthase; *OR*, Orange protein; *OR-like*, Orange-like protein.

Transcription factors and regulatory genes potentially affecting lettuce metabolism under varying light and temperature regimes were identified based on the literature (Li et al., 2010; Stracke et al., 2010; Toledo-Ortiz et al., 2014; Stanley and Yuan, 2019). Selected genes encoding key enzymes of the core biosynthesis for carotenoid (Phytoene synthase, *PSY*) and flavonoid pathways (Chalcone synthase, *CHS*) were analyzed as *n*-fold expression based on lettuce cultivation without polytunnels (**Figure 5**) to obtain further insights into their metabolic regulation.

The *CHS* gene encodes an enzyme early in the flavonoid pathway (**Figure 1**) that catalyzes the condensation of cinnamic acid (derivative) CoA-ester with malonyl-CoA yielding naringenin chalcone (Dao et al., 2011). It is thus the hub for the synthesis of a wide diversity of flavonoids in plants. The expression of *CHS* was 14.47-fold lower in lettuce grown under polytunnels with antifog, and 11.34-fold lower under polytunnels without antifog than lettuce grown without polytunnels. However, only a trend can be detected in the lower *CHS* expression under polytunnels without antifog. In agreement with this observation, the transcripts of *UVR8* (UV resistance locus 8) are also 2.05-fold lower in both polytunnels compared to cultivation without polytunnels. This gene encodes the *Arabidopsis* UVB photoreceptor, which is known to induce UVB light-triggered metabolic responses (Rizzini et al., 2011).

The enzyme encoded by the *PSY* gene is the first committed step in carotenoid biosynthesis (Von Lintig et al., 1997). *PSY* expression in lettuce was 1.52-fold lower when grown under polytunnels (independently of antifog) than without polytunnels. This is a relevant observation, since the amounts of carotenoids are significantly higher in lettuce grown under polytunnels. In contrast, the transcripts of *CCD4* (Carotenoid dioxygenase 4), a gene encoding a cleavage enzyme that forms apocarotenoids, were 5.02-fold higher. Taken together, fewer transcripts for the *PSY-*based biosynthesis, and more abundant transcripts for the *CCD4*-based degradation, together with contrasting higher amounts of carotenoids suggest that additional mechanisms are important for regulating the carotenoid pool in lettuce. *OR* and *OR-like* encoding proteins, known as posttranscriptional regulators (Zhou et al., 2015), interact with PSY. In lettuce grown without or under polytunnels, no differences were observed for either *OR* or *OR-like* transcripts.

The transcription factors *HY5* (Elongated hypocotyl 5) and *PIFs* (Phytochrome interacting factors), are closely related to light and temperature signaling and impact metabolic responses of both carotenoids and flavonoids (Lee et al., 2007a; Toledo-Ortiz et al., 2014; Stanley and Yuan, 2019). Here, *HY5* and *PIF1* (Phytochrome interacting factor 1) are antagonistic: while *HY5* acts as transcriptional activator and is able to bind to *CHS* and *PSY* promotors, *PIF1* acts as transcriptional repressor and is able to bind the *PSY* promotor. This highlights *HY5* and *PIF1* as promising candidates to be investigated. In lettuce grown without or under polytunnels, *HY5* transcripts were similar and no significant differences were evident, whereas *PIF1* transcripts were 1.30-fold higher in lettuce under polytunnels. The experimental repetitions also showed predominantly similar patterns, although in some cases tendencies are present (**Figures S10** and **S11**). For example, *PSY* expression is lower in repetitions 2 and 3 in polytunnel grown lettuce, albeit not significantly; however, the accumulation of carotenoids in such lettuce still cannot be explained.

In summary, the transcripts of flavonoid-related biosynthetic enzymes were associated with flavonoid content in lettuce. Transcripts of UVB photoreceptor *UVR8* were lower in lettuce grown under polytunnels, and thus related to flavonoid content. However, the transcripts for carotenoid-related biosynthetic enzymes did not show this kind of association with carotenoid content. Focusing on the transcription factors, *HY5* transcription did not seem to differ in any experimental repetitions, whereas the transcription level of *PIF1* was higher in lettuce grown under polytunnels than without polytunnels.

### 3.5 Polytunnel cultivation affects the phytohormone abscisic acid

**Figure 6:**
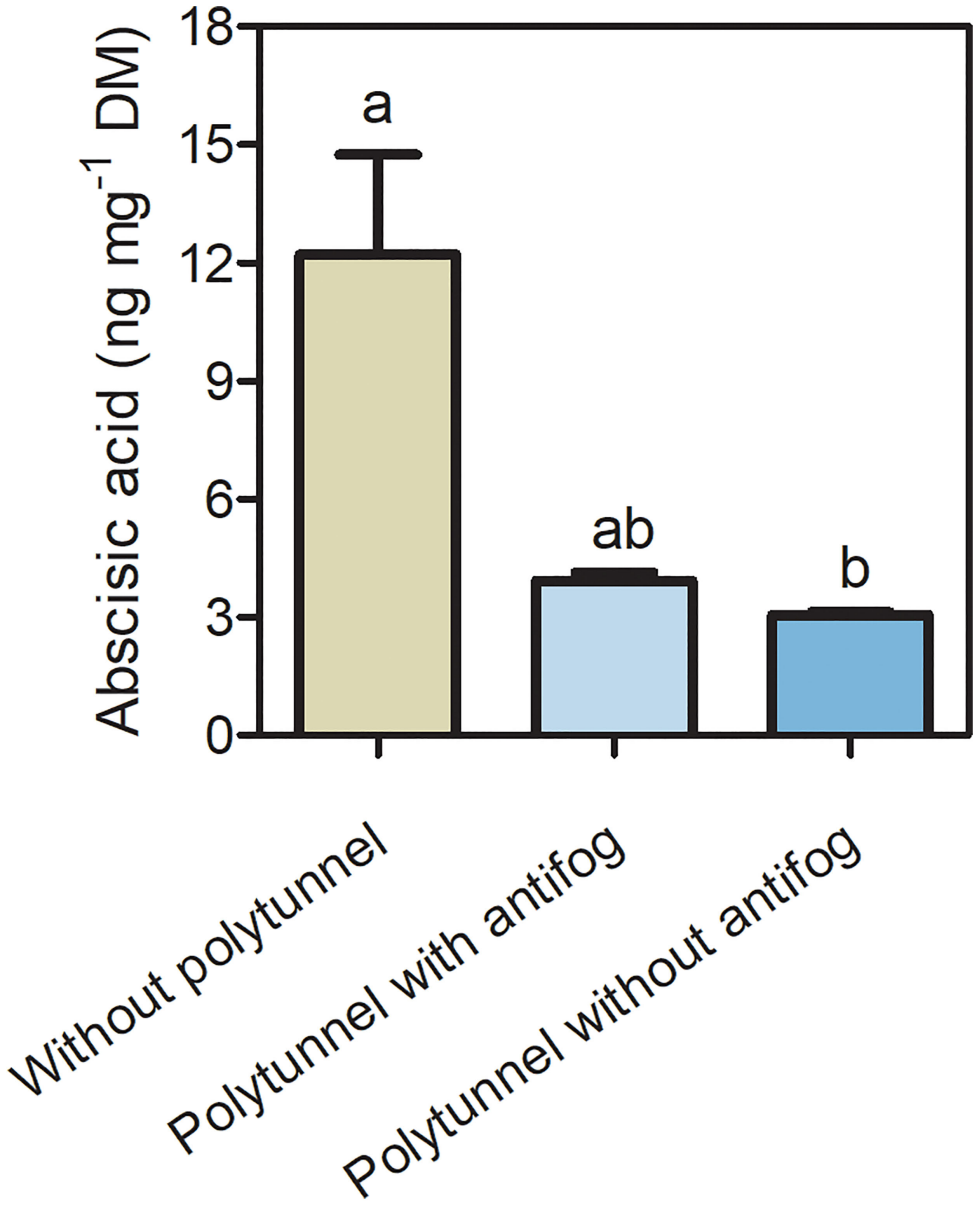
Phytohormone ABA content in lettuce. Abscisic acid content (ng mg^-1^ DM) in lettuce grown without or under polytunnels with and without antifogging additives. The first experimental repetition in April is shown. The data are expressed as mean ± SE (n = 4). Different letters indicate significant differences (p ≤ 0.05).

Since phytohormones act as signal transducers and are responsive to changing environmental conditions, we analyzed the content of the phytohormone ABA (**Figure 6**). A 4.01-fold lower ABA content was determined in lettuce under polytunnels without antifog compared to without polytunnels. A tendency of lower ABA content (3.11-fold) was observed when comparing polytunnels with antifog to without polytunnel. Similarly, in the third, but not the second repetition, ABA in lettuce was significantly lower due to polytunnel cultivation (**Figure S12**).

### 3.6 Polytunnel cultivation alters carotenoid metabolic flux

**Figure 7:**
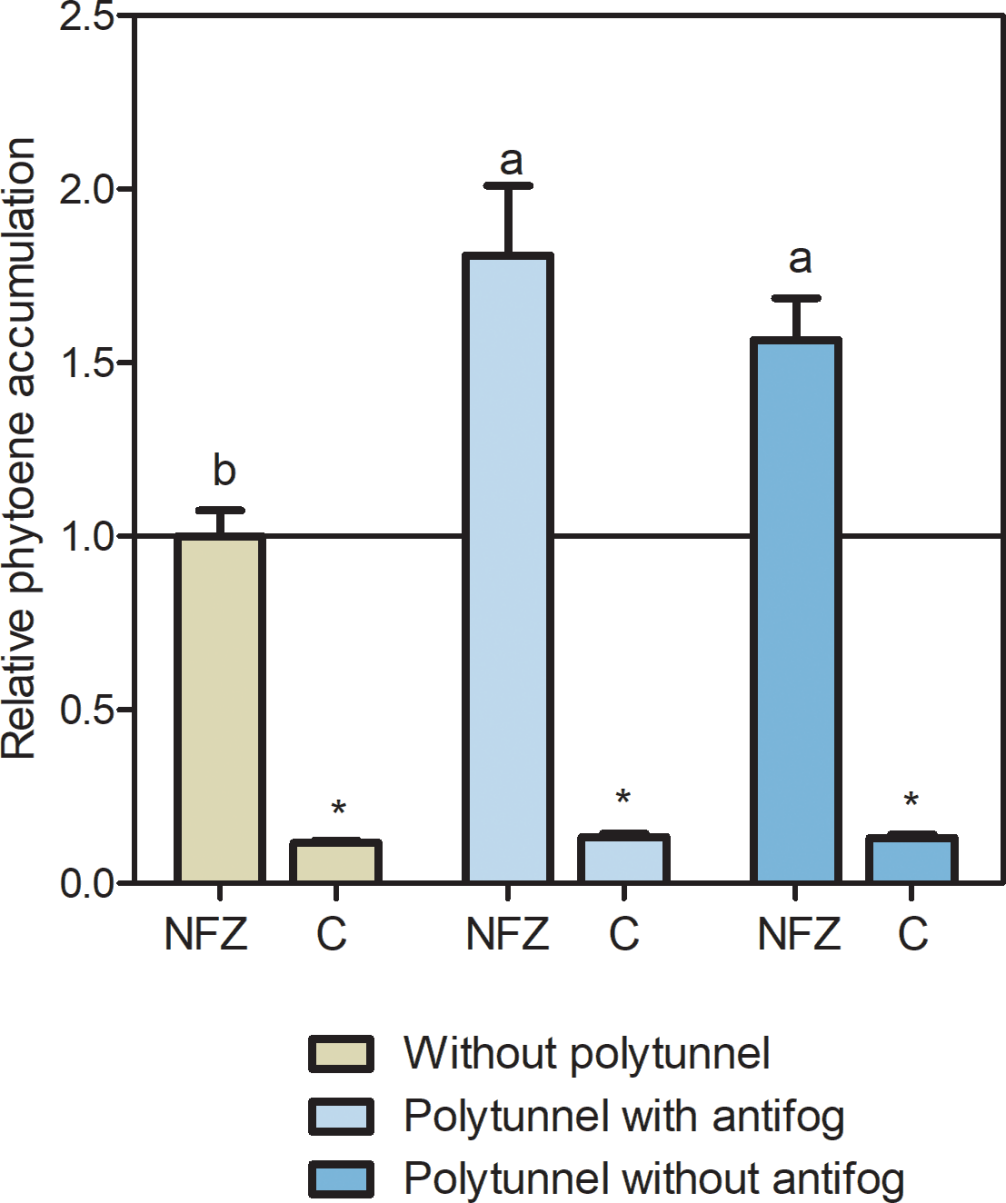
Metabolic flux of the carotenoid pathway determined with norflurazon. Relative phytoene accumulation in lettuce grown without or under polytunnels with and without antifogging additives. Lettuce was treated with the bleaching herbicide norflurazone (NFZ) or with water as a control **(C)**. The data are expressed as mean ± SE of two independent experimental repetitions (each with n = 4). Data were normalized to NFZ lettuce cultivation without polytunnels (indicated by the black line). Asterisks indicate significant differences of NFZ treatment to control (p ≤ 0.05), whereas different letters indicate significant differences due to cultivation condition (p ≤ 0.05).

Since neither transcript levels of *PSY*-based carotenoid synthesis nor *CCD4* pathway-based degradation can explain the carotenoid accumulation in lettuce cultivated under polytunnels, we looked more closely at the carotenoid biosynthetic pathway. Therefore, we investigated the metabolic flux of the upper carotenoid pathway using the bleaching herbicide norflurazon. This inhibits carotenoid pathway enzyme PDS and those downsteam, resulting in phytoene accumulation, and indicating metabolic flux (Koschmieder and Welsch, 2020). Overall, norflurazon treatment led to higher amounts of phytoene in lettuces than in the water treated controls, independently of cultivation conditions (**Figure 7**). In lettuce grown without polytunnels, phytoene amounts were 8.53-fold higher, whereas in lettuce under polytunnels with antifog they were 13.45-fold higher, and without antifog 11.87-fold higher than the water controls. Notably, under norflurazon treatment higher (1.69-fold) phytoene contents were determined in lettuce grown under polytunnels than without, indicating higher carotenoid metabolic flux. This provided a possible explanation for the higher carotenoid contents.

## 4 Discussion

Growing crops under protected cultivation leads to differing climatic conditions compared to open-field production. Furthermore, the covering materials used can affect these conditions. The polytunnels used in this study influenced UV and PAR light regimes as well as temperature. Although reduced transmission was determined in this range, there were no changes in the ratio of far-red and red light. The generated greenhouse effect resulted in temperature increases closely related to the modulated light conditions. Polytunnels with and without antifogging additives showed no differences in temperature nor UV transmittance, and only minor differences in PAR intensity. For both polytunnel covering materials, with and without antifog, similar differences in metabolite profiles, transcripts, phytohormones, and metabolic fluxes were observed. Consequently, the results are not discussed individually but are summarized below under the general term ‘polytunnel’.

### 4.1 Regulation of flavonoid biosynthesis in protected cultivation

Flavonoids are bioactive compounds from the diverse group of plant phenols and based on structural properties are divided into subgroups such as the flavonols and flavones, both detected in lettuce. In terms of human consumption, epidemiological studies have shown that flavonoid-rich foods have beneficial properties, for instance against chronic metabolic or cardiovascular diseases (Hertog et al., 1993; Kim and Je, 2017; Rees et al., 2018). In plants, they are known as antioxidants capable of scavenging reactive oxygen species (ROS) produced for example under solar UV light exposure (UVA and UVB) or plant stress (Agati and Tattini, 2010). Thus, the UV-shielding flavonoids are mainly found in the plant epidermis and act as UV protectants – a plant’s sunscreen. Studies have reported that flavonoid contents increase in lettuce grown with additional UVA, UVB and UVC light (Lee et al., 2014; Assumpção et al., 2019).Using covered systems for crop cultivation, changes in UV light occur due to the reduced transmittance of the materials used. Indeed, UVA and UVB light are reduced by using polytunnels in this study, resulting in lower total and individual flavonoids than seen in cultivation without polytunnels. This is in accordance with Katsoulas et al. (2020), who reviewed that UV-blocking materials, defined as complete or partial solar UV absorbing materials, have negative effects on plant phenolic compounds such as flavonoids, including in lettuce.

Transcription of the *CHS* gene, encoding a key enzyme in the flavonoid pathway, is reported to be induced by UVA and UVB light (Jenkins et al., 2001; Dao et al., 2011). Accordingly, lettuce cultivated under polytunnels showed lower *CHS* expression than cultivation without polytunnels. The *CHS* promotor contains multiple light regulatory units, and photoreceptor signaling may play a role in the interaction with such units. In *Arabidopsis*, there is evidence that solar UV in particular is able to trigger changes in the flavonoid biosynthetic pathway *via* UVR8 photoreceptor regulation together with other photoreceptors, where UVB specifically triggers a UVR8 response (Morales et al., 2013). We assume the UVR8 photoreceptor contributes to flavonoid regulation in lettuce grown under polytunnels; however, the UVB signal transduction pathway from UVR8 to flavonoid biosynthesis is not well understood (Yang et al., 2018). The UVB/*HY5* pathway has been well studied. *HY5* is known to bind in promotor regions of several genes, such as *CHS* (Lee et al., 2007a; Toledo-Ortiz et al., 2014) and so can act as a transcriptional activator. Stracke et al. (2010) showed that *HY5* is required in light and UVB responsive gene regulation. However, in this study, lettuce grown without or under polytunnels showed no significant differences in *HY5* transcripts in any experimental repetition. Consequently, regulation may occur at the protein level, which should be clarified in further studies. Furthermore, interactions with other transcription factors, for example from the MYB family, have been described before (Cloix and Jenkins, 2008; Luo et al., 2008; Qian et al., 2020)

In addition to transcriptional induction of flavonoid accumulation, studies suggest that ABA has an impact on regulating flavonoid biosynthesis. ABA is a phytohormone that affects plant development and growth, among other things, and also acts as signaling molecule in response to abiotic and biotic environmental conditions (Vishwakarma et al., 2017). For example, Berli et al. (2010) showed increased flavonoid levels in leaves of *Vitis Vinifera* L. when exogenous ABA was applied, whereas another study also shows involvement of *PAL* (Phenlylalanine ammonia-lyase) enzyme activity (Hao et al., 2010). In the present study, higher amounts of flavonoids related to higher ABA content were found in lettuce grown without polytunnels than grown in polytunnels. Altered ABA levels are in particular discussed in response to high light, UVB irradiation or changing temperatures (Vishwakarma et al., 2017; Brunetti et al., 2019). Furthermore, it has been shown that in leaves of *Vitis Vinifera* L. less ABA and flavonoids accumulated when UVB light is filtered by a polyester covering (Berli et al., 2010). It is worth noting that *HY5* transcriptional activation is also discussed as contributing to ABA signaling (Chen et al., 2008). Thus, the role of *HY5* in lettuce without and with polytunnels remains uncertain.

Neugart et al. (2019) demonstrated a priming effect of the PAR light regime in the response of *Arabidopsis* to additional UVA and UVB light. Therefore, the accumulation of flavonoids could be influenced by both light regimes in this study.

In addition to the light regime, temperature is another regulating factor related to flavonoid content. Studies on the effect of temperature on flavonoids in lettuce are contrasting; while most studies show higher flavonoid accumulation in lettuce grown at lower temperatures (Boo et al., 2011; Sytar et al., 2018), one study shows the opposite (Sublett et al., 2018). In other plant species such as *Arabidopsis thaliana*, *Ginkgo biloba* L. or *Angelica sinensis*, the flavonoid accumulation was promoted at lower temperatures (Leyva et al., 1995; Guo et al., 2020; Dong et al., 2022). This supports the higher flavonoid accumulation in this study, in which temperatures were between 1.49°C to 3.13°C lower when lettuce was grown without polytunnels. In addition, transcript levels of key biosynthetic enzymes like *PAL* or *CHS* as well as enzyme activity were shown to be highest at lower temperatures (Leyva et al., 1995; Guo et al., 2020; Dong et al., 2022). This could lead to the assumption that the *CHS* transcripts and flavonoid content in lettuce in this study were possibly also related to the temperature regime. However, the study by Sytar et al. (2018) showed that this should be taken with caution. They observed that UV light, but not cultivation temperature, had the major impact on flavonoid accumulation in lettuce grown in greenhouses and outdoors (Sytar et al., 2018).

Lettuce grown under polytunnels contains lower total and individual flavonoid compounds, mainly due to the reduced solar UV (UVA and UVB) transmissibility of the covering material. This is also reflected in the transcript levels of biosynthetically active genes, which are regulated at the transcriptional level.

### 4.2 Regulation of carotenoid biosynthesis in protected cultivation

As accessory pigments, carotenoids are involved in light harvesting and contribute to effective photosynthesis. Additionally, they are also involved in non-photochemical quenching (NPQ) and protect plants from adverse environmental conditions such as excessive light or high temperatures (Cazzonelli, 2011). In human nutrition, carotenoids are associated with several health-promoting properties, such as a lower risk of non-transmissible diseases or protection against age-related macular degeneration (Eisenhauer et al., 2017; Milani et al., 2017). Thus, there is consumer interest in vegetables rich in these bioactive compounds, and understanding their metabolic regulation is important for targeting their enhancement.

The carotenoid pathway in plants is highly conserved and well understood (Stanley and Yuan, 2019); however, regulatory mechanisms are still the subject of research. Since the regulation of carotenoids appears to be species- and tissue-specific (Ohashi-Kaneko et al., 2007; Shumskaya et al., 2012; Lätari et al., 2015), and plant research mainly focuses on model plants, such as *Arabidopsis thaliana*, little is known about its regulation in horticultural crops. In lettuce grown under polytunnels, we observed higher total and individual carotenoids compared to cultivation without polytunnels.

The PSI and PSII photosystems have different absorption maxima due to their carotenoid and chlorophyll composition, possibly leading to different adaptations of their composition depending on both light quality and quantity (Ballottari et al., 2007; Caffarri et al., 2014). A carotenoid steady-state has been suggested as a balance between biosynthesis and turnover in photosynthetic leaf tissue (Lätari et al., 2015). The adaptation of carotenoid content and profile in leaves probably results from an imbalance in the photosystem’s excitation, as suggested by Frede et al. (2019) for pak choi (*Brassica rapa* subsp. *chinensis*) sprouts illuminated with different LED light qualities. Changing environmental conditions can cause the photosynthetic pigments to adapt and achieve effective light harvesting that contributes to photosynthesis. Altered light quality due to the reduction in the UV/PAR and to a lesser extent far-red/red wavelength by the covering material, as well as altered light intensity, occur in polytunnel cultivation. As part of the photosynthetic apparatus, carotenoid changes are concomitant with changes in chlorophylls, and co-expression of chlorophyll- and carotenoid-related genes is also evident (Meier et al., 2011; Stange and Flores, 2012).

In this study, increased carotenoid and chlorophyll contents were observed in polytunnel grown lettuce compared to without polytunnels for all three experimental repetitions (**Figures S13**-**15**). In particular, the decrease in chlorophyll a/b ratio indicates an adaptation of the photosystems and alteration in chlorophyll metabolism to achieve effective light harvesting under polytunnels, since chlorophyll b acts as an accessory pigment in the antenna (Lee et al., 2007b; Caffarri et al., 2014). This is also reflected in individual carotenoids. Depending on their localization and function in the photosystems, β-carotene and zeaxanthin in core structure, and lutein, violaxanthin and neoxanthin in light harvesting antenna (Caffarri et al., 2014), one can suggest that the lower light intensity and altered spectral quality under polytunnels leads to adaptation of accessory carotenoids. Besides the light regime, it is discussed that carotenoids protect plants at high temperatures by scavenging resulting in reactive oxygen species (ROS) generated in PSII and thylakoid membranes (Stanley and Yuan, 2019). Toledo-Ortiz et al. (2014) determined higher carotenoid contents in *Arabidopsis* grown at higher temperatures (17°C to 27°C), while there was only a positive trend in kale and spinach (from 10°C to 30°C) (Lefsrud et al. (2005). This is consistent with the higher carotenoids in lettuce and the prevailing polytunnel conditions in this study, although the major antioxidant carotenoids in the photosystem core structure, β-carotene and zeaxanthin, appear to be less responsive in lettuce.

Studies demonstrate that higher *PSY* transcription is related to carotenoid accumulation induced by changes in light regimes (Von Lintig et al., 1997; Frede and Baldermann, 2022). Furthermore, *PSY* transcription was shown to be lower at elevated temperatures in maize leaves, while carotenoid accumulation was higher (Li et al., 2008). For this reason, it is assumed that the *PSY* transcript is not responsible for regulating carotenoid metabolism at higher temperatures (Stanley and Yuan, 2019). The synthesis of phytoene *via* PSY is a key and rate-limiting step in carotenoid biosynthesis. Surprisingly in this study, the *PSY* transcripts in lettuce predominantly showed no major differences or decreases in polytunnel cultivation, which are not reflected in the lettuce carotenoid contents and may indicate temperature-dependent regulation. How plants regulate the carotenoid biosynthetic pathway, particularly under different light and temperature regimes, is still largely unknown. Nevertheless, the involvement of some essential transcription factors has been confirmed (Stanley and Yuan, 2019). *HY5* functions as an intermediate control point downstream of photoreceptor signal transduction (Toledo-Ortiz et al., 2014). In light, *HY5* can bind to the *PSY* promotor and *HY5* accumulation is stabilized at lower temperatures favoring the binding to the Arabidopsis *PSY* promoter (Toledo-Ortiz et al., 2014). However, in this study, involvement of *HY5* in the regulation of carotenoid biosynthesis is unlikely since *HY5* transcripts showed no differences in lettuce grown without or with polytunnels. Moreover, the *HY5* antagonist *PIF1* showed higher transcript levels in the investigated lettuce.

*PIF1* antagonizes *HY5* by binding to the same target at *PSY* promotor and repressing its transcription in the dark and elevated temperatures (Toledo-Ortiz et al., 2014). In de-etiolated leaves of *Arabidopsis* and *Sinapis alba*, *PSY* transcription seems to be phytochrome mediated *via PIF1* (Von Lintig et al., 1997). Moreover, *PIF1* is involved in shade-triggered reduction of carotenoid accumulation through *PSY* modulation in a *HY5* independent manner (Bou-Torrent et al., 2015). Both processes are triggered by far-red to red light, among others. Apart from carotenoid regulation, *PIF1* is found to interact with chlorophyll biosynthetic genes (Moon et al., 2008), and thus the regulation of carotenoids and chlorophylls are closely linked to photosynthetic efficiency. Furthermore, carotenoid and chlorophyll accumulation in *Arabidopsis* pif1 mutants were most affected at elevated temperature (Toledo-Ortiz et al., 2014). Therefore, the observed *PIF1* accumulation in this study results from low light, altered light qualities and elevated temperatures in polytunnels (Toledo-Ortiz et al., 2014). We assume that lettuce in polytunnels accumulates less *PSY* transcripts through *PIF1* triggered repression related to light and temperature regimes. In contrast, carotenoid contents are increased, suggesting additional mechanisms beyond the transcriptional level. Therefore, further research should aim to elucidate the regulation by the HY5/PIF1 network at the protein level.

The OR protein family is known from yellow cauliflower and its involvement in carotenoid accumulation (Lu et al., 2006). OR family members *OR* and *OR-like* are known to act in posttranscriptional regulation with PSY in leaves (Zhou et al., 2015). In this study, lettuce transcript levels of both were elevated in polytunnel cultivation in the second but not the first or third experimental repetition (**Figures 5**, **S10**, **S11**). Thus, *OR*-derived posttranscriptional regulation based on *OR* and *OR-like* transcripts do not explain carotenoid accumulation.

The carotenoid content is not only altered by biosynthesis, but also by degradation. CCDs (carotenoid cleavage dioxygenases), characterize a group of carotenoid-degrading enzymes. Recent studies indicate an effect of light quality on *CCD4* transcription Frede et al. (2018). Furthermore, a more recent study by Frede and Baldermann (2022) showed that a combination of blue and white light leads to both increased *CCD4* transcript levels and carotenoid content. We observed increased transcription levels of *CCD4*, which indicates higher carotenoid turnover as suggested by Frede and Baldermann (2022).

Since carotenoid metabolic flux was found to be higher in lettuce grown under polytunnels than without, carotenoid accumulation is likely independent of *PSY* transcription. Therefore, PSY protein levels or PSY enzyme activity could be different due to cultivation conditions. Here, different mechanisms are known in other species. Firstly, posttranscriptional regulation of PSY involving phytochrome photoreceptors seems reasonable. This has been discussed in tomato fruit: specifically, PSY enzyme activity but not transcription levels were different in tomatoes grown in red light grown compared to red/far-red and the dark (Schofield and Paliyath, 2005). Secondly, PSY localization appears to be important for its enzyme activity, particularly observed with regard to light qualities (Welsch et al., 2000). The membrane-bound PSY protein is active and contributes to metabolic flux, whereas the soluble PSY protein in the stroma is inactivate (Shumskaya et al., 2012; Lätari et al., 2015). Thirdly, light and temperature differences can cause changes in membrane fluidity, which were discussed for light or heat stress by Yamamoto (2016), and might contribute to the solubility of PSY protein. In order to shed light on posttranscriptional mechanisms of lettuce carotenoid accumulation, it is essential to analyze protein levels and enzyme activities in future research. Since PSY is affected by light in the red and far-red regions, special emphasis should also be given on greenhouse films in these light regions and their effects on the regulation of these bioactives.

Finally, as discussed above for flavonoids, ABA might be involved in mediating carotenoid steady-state levels. In *Arabidopsis*, induction of *PSY* transcription at post-germination under continuous light is negatively regulated by ABA (Meier et al., 2011). However, in the present study, *PSY* transcripts are reduced or unaffected and are probably not predominantly affected by ABA. Exogenous application of ABA shows contrasting effects depending on plant tissue and species (Baldermann et al., 2013; Barickman et al., 2014; Liu et al., 2020). Overall, no conclusion can be made about ABA being involved in mediating carotenoid accumulation.

Polytunnel cultivation of lettuce leads to higher total and individual carotenoids, mainly related to altered spectral quality and light intensity due to the covering material and higher temperatures. *PIF1* transcripts are associated with biosynthetically active genes, suggesting an influence of phytochromes. Nevertheless, the higher carotenoid content seems more likely to be the result of higher metabolic flux due to posttranscriptional regulation of PSY. Further research should aim to elucidate the underlying mechanism influencing PSY but also CCD4 protein amounts as well as their activities.

### 4.3 Co-regulation of carotenoid and flavonoid pathways

Carotenoids and flavonoids both share similar functions in plants as protective compounds, as antioxidants scavenging ROS to protect the photosynthetic apparatus from photoinhibition (Agati and Tattini, 2010; Cazzonelli, 2011). Hence, they are involved in maintaining efficient plant photosynthesis even under unfavorable environmental conditions. However, in contrast to flavonoids, carotenoids are directly involved in the light harvesting process of photosynthesis, which is reflected in their location in plants. Carotenoids are bound to membranes in the chloroplast, whereas flavonoids primarily accumulate in the epidermis but also in chloroplasts’ envelope to quench ROS (Agati and Tattini, 2010; Cazzonelli, 2011). For this purpose, these bioactive compounds have light-absorbing structures; however, their structural properties result in different light absorption characteristics. Carotenoids absorb predominantly in the blue light region (about 450 nm), whereas flavonoids absorb in the UV region (about 350 nm). Thus, by filtering UV light, flavonoids shield chloroplasts and the photosynthetic apparatus from UV-induced ROS production. Quercetin and luteolin flavonoids, both predominant in lettuce, in particular are discussed to efficiently protect plants due to their catechol structure in their B-ring, although the glycosylation pattern also affects these properties (Agati and Tattini, 2010; Neugart et al., 2019).

In the presented study, we observed an inverse relationship between carotenoids and flavonoids. Lettuce cultivated under polytunnels has higher carotenoid but lower flavonoid content, whereas in lettuce cultivated without polytunnels the findings are the opposite. This was also seen in the individual experiments, with the highest correlation found for lettuce grown in May (Spearmans: 0.832, repetition 2) followed by moderate correlations in April (Spearmans: 0.464, repetition 1) and September (Spearmans: 0.459, repetition 3), although no significance was found for the latter two (**Figure S16**). In all experiments, similar effects were found by growing lettuce without or under polytunnels, although the total amounts of both metabolites varied. A moderate negative correlation (Spearmans: 0.406) between carotenoids and flavonoids was also observed when all experimental repetitions were included.

The observed inverse relationship between carotenoids and flavonoids is in accordance with literature, where several studies revealed antagonistic occurrence of carotenoid and flavonoid content within different treatments (Ngwene et al., 2017, Ben Cao et al., 2015; Abdallah et al., 2016; Neugart et al., 2018). For instance, Becker et al. (2015) studied the impact of nitrogen deficiency on different metabolites in red and green lettuce. Here, carotenoids and chlorophylls decreased, while flavonoids increased under a lack of nitrogen.

Despite our initial assumption that *HY5* is involved in the inverse regulation of carotenoids and flavonoids, we hypothesize that there is no underlying regulatory mechanism based on transcriptional control of the biosynthetic pathways, but rather this phenomenon of inverse levels of carotenoid and flavonoid content is physicochemical in nature. Under higher light intensities without polytunnels, the light shielding properties of flavonoids are crucial to protect the plant from excessive UV light, while at lower light intensities under polytunnels the light must be used as efficiently as possible, which can lead to an increase in carotenoids as light harvesting compounds. The inverse relationship between carotenoids and flavonoids could thus be explained by their differing relative importance under different light intensities: high light protection/flavonoids and low light harvest/carotenoids. This is supported by our data, since the correlation is weaker for experiments conducted in months with lower solar radiation, leading to less flavonoid but higher carotenoid accumulation for effective light harvesting.

Further research could aim to elucidate the inverse relationship between the carotenoid and flavonoids by using a metabolic flux analysis with isotopically labeled compounds. Since mechanisms are species specific not only model plants, but also crop plants should be targeted.

### 4.4 Limitations and potential

There are certain limitations when studying the impact of protected cultivation systems. A balance must be found between greenhouse size and replicates as we discussed previously (Harbart et al., 2022). In this study, we evaluated the effects of solar radiation on selected bioactive compounds. We did not use supplementary artificial light, but this is likely to affect the performance of the antifogging additives: in this study no differences were observed when comparing both greenhouse covering materials, in contrast to our previous study using artificial light (Harbart et al., 2022). Moreover, the polytunnel and greenhouse conditions are not, or only semi-controlled cultivation conditions. This is reflected in the climatic conditions we recorded in experimental repetitions due to seasonal changes (**Table 1**). Photoperiods, DLIs and temperatures differed to some extent. Despite the experimental variations, similar results (tendencies) were found for most metabolites and changes in transcripts. Nevertheless, there were some differences. In particular, ABA content was different in the second experimental repetition: here no differences were observed between lettuce grown without or under polytunnels. We assume this is likely due to a rapid temperature increase a few days before harvesting (**Figure S17**). Conducting such experiments under controlled conditions as in a phytochamber is hardly possible due to the required size and number of polytunnels. However, limiting slightly varying conditions in the experimental repetitions would potentially make the observed effects more consistent and robust.

## 5 Conclusion

Bioactive compounds such as carotenoids and flavonoids have health-beneficial properties when integrated into the human diet. Understanding the regulatory mechanisms of their biosynthetic pathways in differently cultivated horticultural crops is crucial for optimizing conditions for nutrient-rich crops. The aim of this study was to examine the effects of varying climatic conditions in protected cultivation in lettuce. Covering materials impacted light quality and quantity in close relationship to the temperature determined under the polytunnels compared to without polytunnels. Flavonoid contents decreased whereas carotenoid contents increased, showing an inverse correlation, although all antioxidants, the regulatory mechanisms responsible for their accumulation were found to be different. Flavonoid accumulation in lettuce appears to be predominantly regulated by solar UV light detection at a transcriptional level, whereas the carotenoid steady-state levels are regulated posttranscriptionally. In conclusion, the production of nutrient-rich horticultural crops has to be balanced between various influential factors favoring accumulation of health-beneficial compounds under protected cultivation, such as season, location or type and composition (e.g. UV- or red/far-red- blocking/UV- or red/far-red- transmissible) of agricultural films.

## Data availability statement

The original contributions presented in the study are included in the article/**Supplementary Material**. Further inquiries can be directed to the corresponding author.

## Author contributions

VH and SB designed the research. VH accomplished experiments. HPLC analysis of ABA and evaluation was performed by VH with the help MF and RT-*q*PCR analysis was performed by VH with the help of KF. VH drafted the manuscript. Revision of the manuscript was done by MF, KF and SB. All authors contributed to the article and approved the submitted version.
