# Supplementary material for: Regulation of carotenoid and flavonoid biosynthetic pathways in Lactuca sativa var capitate L. in protected cultivation
Source: Front Plant Sci. 2023 Feb 14;14:1124750. doi: 10.3389/fpls.2023.1124750 (PMC9971571; doi:10.3389/fpls.2023.1124750)
Supplement: Supplementary file 1 [file DataSheet_1.pdf]

# *Supplementary Material*

**Supplementary Table S1.** Parameters for flavonoid glycoside identification in lettuce. Compounds verified with authentic standards are marked with †.

| Compound name | Retention time (min) | Ion | MS¹ *m/z* | MS² *m/z* | MS³ *m/z* | Absorption maxima (nm) |
|---|---|---|---|---|---|---|
| Quercetin-3-glucuronide† | 16.10 | $[M-H]^-$ | 477 | 301 | 257 179 | 222 256 352 |
| Quercetin-3-malonylglucoside† | 19.56 | $[M-H]^-$ | 549 505 | 463 301 | 257 179 | 256 354 |
| Luteolin-7-glucuronide† | 18.53 | $[M-H]^-$ | 461 | 285 | | 222 252 346 |

Identification based on the literature: Becker, C. et al., 2015: PLoS One 10, 11 e0142867, Llorach R et al., 2008: Food. Chem. 108(3), 1028-1038.

**Supplementary Table S2.** Parameters for carotenoid and chlorophyll identification in lettuce. Compounds verified with authentic standards are marked with †. Unless otherwise stated, carotenoids have *all-trans* stereochemistry.

| Compound name | Retention time (min) | Ion | MS *m/z* | Absorption maxima (nm) |
|---|---|---|---|---|
| β-Carotene† | 44.21 | $[M+H]^+$ | 537.44 | 426 452 478 |
| Lutein† | 18.30 | $[M+H-H_2O]^+$ | 551.43 | 420 445 472 |
| Lactucaxanthin | 16.21 | $[M+H-H_2O]^+$ | 551.43 | 414 438 468 |
| Zeaxanthin† | 20.02 | $[M+H]^+$ | 569.43 | 424 450 478 |
| Antheraxanthin† | 15.93 | $[M+H]^+$ | 585.43 | 420 442 470 |
| Violaxanthin† | 9.52 | $[M+H]^+$ | 601.42 | 414 438 468 |
| Violaxanthin unidentified isomer | 12.27 | $[M+H]^+$ | 601.43 | 422 447 |
| Neoxanthin (9-*Z*)† | 10.74 | $[M+H-H_2O]^+$ | 583.41 | 412 434 464 |
| Neoxanthin unidentified isomer 1 | 7.94 | $[M+H-H_2O]^+$ | 583.42 | 412 436 466 |
| Neoxanthin unidentified isomer 2 | 8.59 | $[M+H-H_2O]^+$ | 583.42 | 398 420 446 |
| Chlorophyll a† | 21.12 | $[M+H]^+$ | 893.54 | 432 |
| Chlorophyll b† | 17.03 | $[M+H]^+$ | 907.52 | 468 |

| | | | | |
|---|---|---|---|---|
| Phytoene (*E*/*Z*)[†] | 20.59 | $[M+H]^+$ | 545.51 | n.d. |

Identification based on the literature: Diop Ndiaye, N. et al., 2011: J. Agric. Food Chem. 59(22), 12018-12027, Britton, G. et al., 2004: Carotenoids: Handbook, Birkhäuser, Gopal et al., 2017: Food Funct. 8, 1124.

**Supplementary Table S3.** Primer pairs, amplicon size and experimentally determined amplification efficiency (E) used for the gene expression analysis with RT-qPCR.

| Protein (gene name) | Primer pairs (5' to 3') | | E (%) | Amplicon size (bp) |
|---|---|---|---|---|
| Target genes | | | | |
| Phytoene synthase (*PSY*) | F: TATTGTTATTATGTGGCTGGAAC | R: GCATCTTCTCCCACATCTCT | 100 | 161 |
| Carotenoid dioxygenase 4 (*CCD4*) | F: TGGTTTGAGGTGCCTGGATT | R: GATTGATCACAGGAAACTCCAA | 100 | 241 |
| Chalcone synthase (*CHS*) | F: TCAATGATAACAAAGCGATACATG | R: TGCTCCGGGCATGTCAAC | 100 | 234 |
| UVB-resistance 8 (*UVR8*) | F: CAGCAATCACTACTCCTCC | R: CAATTTGCCTTATTCGCAACC | 92 | 356 |
| Elongated hypocotyl 5 (*HY5*) | F: TTAAAGAAGGAATGGAAAGTGATG | R: CTCTCTCTTGCTTGTTGAGC | 76 | 235 |
| Phytochrome interacting factor 1 (*PIF1*) | F: GAAATCAGCAATGGGCGATG | R: CGTTATTATGAAGGTAGAGGTC | 96 | 263 |
| ORANGE protein (*OR*) | F: GGCTTGTGCTAGGTGTGCT | R: CTCGCCATAGCCATTCCTG | 88 | 174 |
| ORANGE-like Protein (*OR-like*) | F: TTCAGATGATTCAGCCGCGT | R: GTCAGGCATCTCGTTGTCC | 100 | 253 |
| Reference genes | | | | |
| Actin 7 (*ACT7*) | F: AAGGATGCTTATGTAGGAGATG | R: TCTGTTTTCCTTGGGATTTAATG | 77 | 201 |
| Ubiquitin-conjugating enzyme E2 2 (*UBC2*) | F: ATGACACTCCTTGGGATGG | R: CAACATCATAGATTGGACTCC | 81 | 177 |
| Elongation factor 1-alpha (*EF1α*) | F: TGAACAAACGTTCATTCAAATACGC | R: CGATGACTGTGCAGTAGTAC | 86 | 126 |

**Supplementary Table S4.** Gene Bank accession numbers of nucleotide sequences used for the primer design.

| Gene name | Phytozome gene ID | GenBank accession number |
|---|---|---|
| *PSY* | Lsat_1_v5_gn_2_71880 (PAC:38961715) | XM_023911676.1 |
| | Lsat_1_v5_gn_3_67861 (PAC:38918955) | XM_023907827.1 |
| | Lsat_1_v5_gn_6_52200 (PAC:38938321) | XM_023886351.1 |
| *CHS* | Lsat_1_v5_gn_2_76880 (PAC:38960353) | XM_023879789.1 |
| | Lsat_1_v5_gn_2_42860 (PAC:38957201) | XM_023878437.2 |
| *UVR8* | - | XM_023886847.2 |
| *HY5* | Lsat_1_v5_gn_6_22420 (PAC:38939429) | XM_023879513.2 |
| | Lsat_1_v5_gn_5_30441 (PAC:38970748) | XM_023903859.2 |
| *PIF1* | Lsat_1_v5_gn_9_11161 (PAC:38926882) | XM_023877721.2 |
| *CCD4* | Lsat_1_v5_gn_5_64880 (PAC:38963506) | XM_023895898.2 |
| | Lsat_1_v5_gn_1_6181 (PAC:38942668) | XM_023877528.2 |
| | Lsat_1_v5_gn_6_71300 (PAC:38940698) | XM_023876919.2 |
| *OR* | - | XM_023888191.2 |
| *OR-like* | - | XM_023878966.2 |
| *ACT7* | Lsat_1_v5_gn_5_41421 (PAC:38969943) | XM_023911194.2 |
| | Lsat_1_v5_gn_5_42341 (PAC:38970681) | XM_023911199.2 |
| | Lsat_1_v5_gn_5_41281 (PAC:38964955) | XM_023878534.2 |
| *UBC2* | Lsat_1_v5_gn_2_88820 (PAC:38961340) | XM_023917173.2 |
| | Lsat_1_v5_gn_4_177020 (PAC:38934583) | XM_023902571.2 |
| | Lsat_1_v5_gn_3_102101 (PAC:38915680) | XM_023888250.2 |
| *EF1α* | Lsat_1_v5_gn_2_97861 (PAC:38962797) | XM_023895734.2 |
| | Lsat_1_v5_gn_1_53981 (PAC:38947268) | XM_023910869.2 |
| | Lsat_1_v5_gn_9_67180 (PAC:38923710) | XM_023891343.2 |
| | Lsat_1_v5_gn_2_109480 (PAC:38962554) | XM_042899440.1 |

Phytozome database (https://phytozome-next.jgi.doe.gov/info/Lsativa_V8)

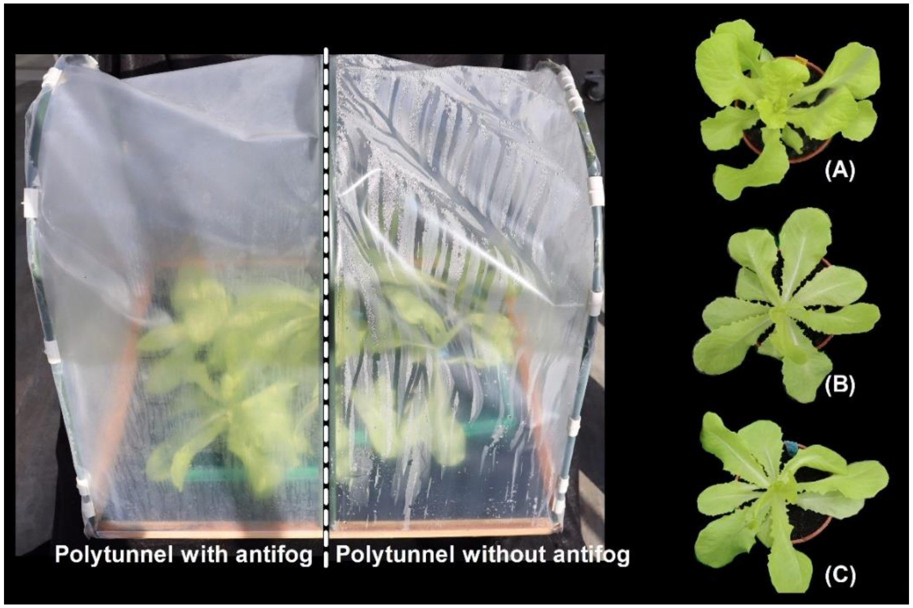


**Supplementary Figure S1 Polytunnel with and without antifogging additives containing lettuce.** (A) Lettuce grown without polytunnel; (B) Polytunnel with antifog; (C) Polytunnel without antifog.

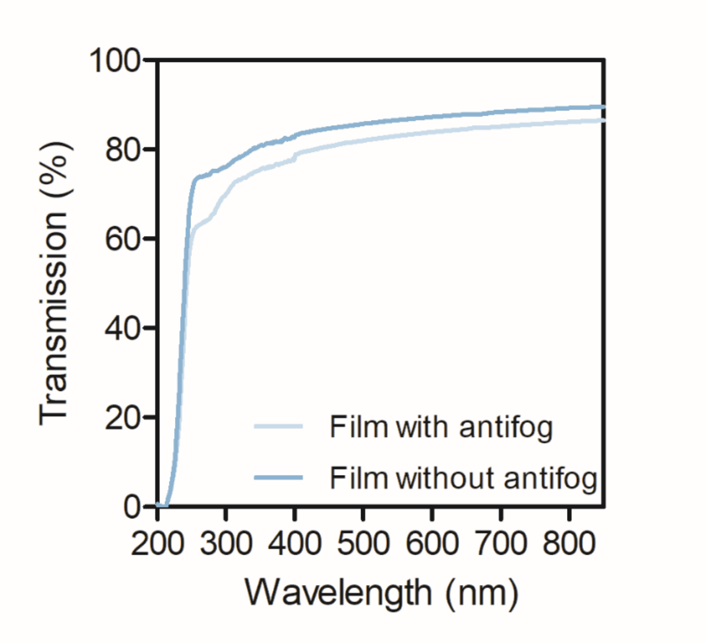


**Supplementary Figure S2 The polytunnel material reduced certain light transmission.** Light transmission spectra of polytunnel films with and without antifogging additives before use.

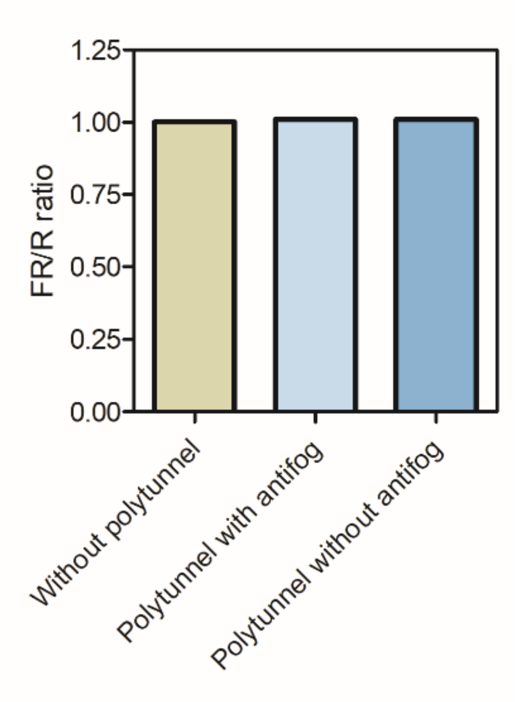


**Supplementary Figure S3 The polytunnel material did not affect far-red to red light ratios.** Far red (FR) to red (R) light ratio of polytunnel films were calculated based on UV/VIS spectral information before use (Figure S2, 655-665 nm and 725-735 nm).

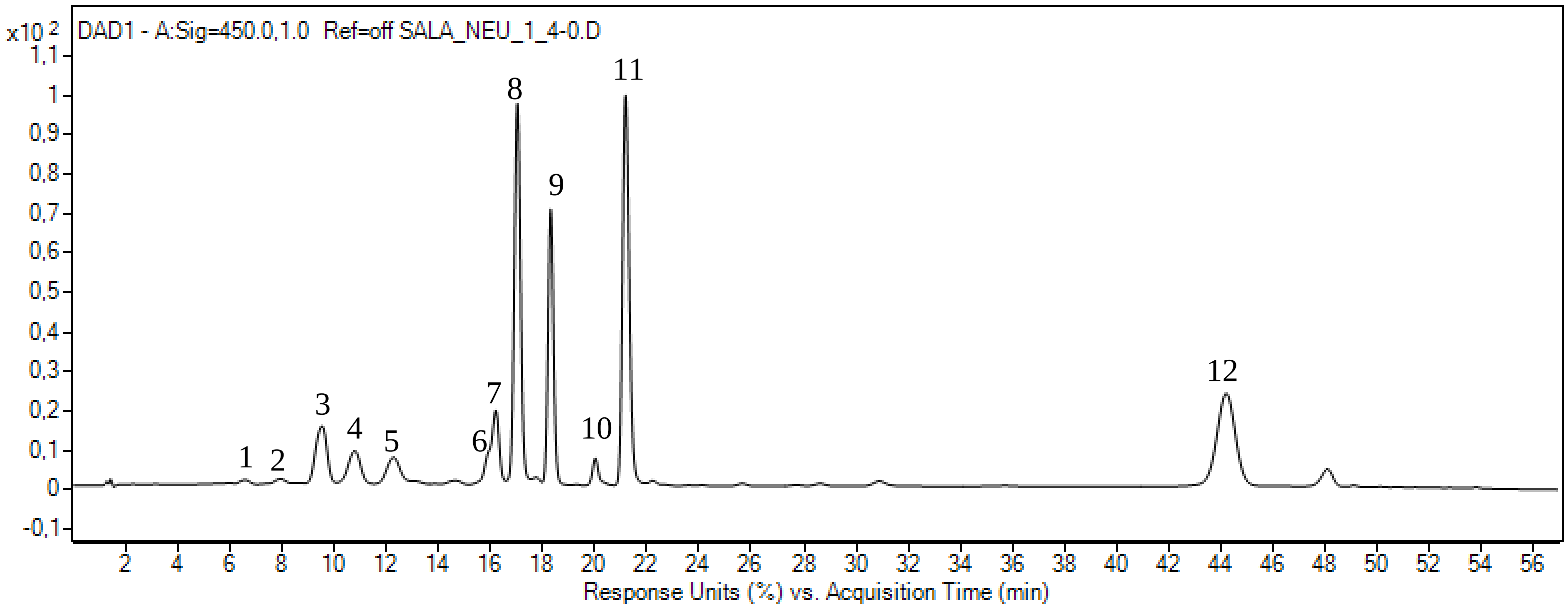


**Supplementary Figure S4 Chromatogram of carotenoids and chlorophylls**. Characteristic chromatogram (450 nm) of carotenoids and chlorophylls in lettuce grown without polytunnels detected with HPLC-DAD-ToF-MS. Unless otherwise stated, carotenoids have *all-trans* stereochemistry. (1) Unidentified neoxanthin isomer 1; (2) Unidentified neoxanthin isomer 2; (3) violaxanthin; (4) neoxanthin (9-*Z*); (5) violaxanthin unidentified *cis*-isomer; (6) antheraxanthin; (7) lactucaxanthin; (8) chlorophyll b; (9) lutein; (10) zeaxanthin; (11) chlorophyll a; (12) β-carotene.

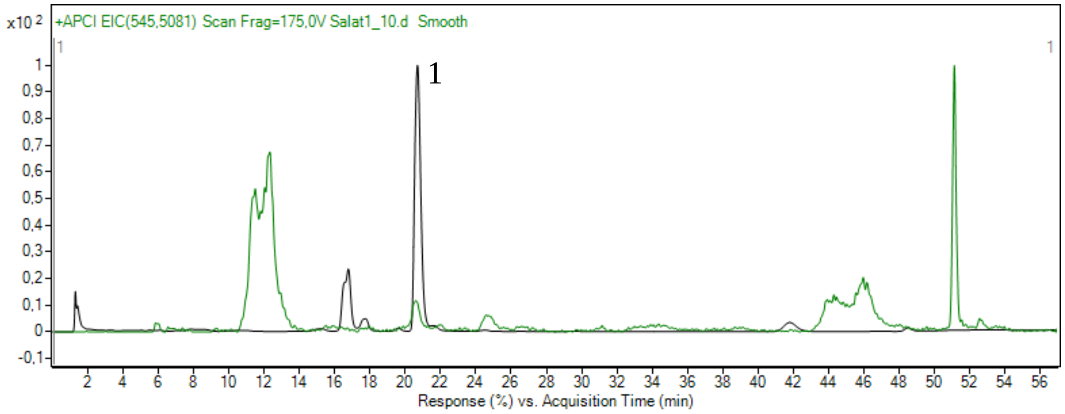


**Supplementary Figure S5 Chromatogram of phytoene**. Characteristic DAD- (280 nm; black) and EIC (*m/z* 545.5081; green) chromatogram of phytoene in lettuce grown without polytunnels detected with HPLC-DAD-QToF-MS. (1) Phytoene isomer.

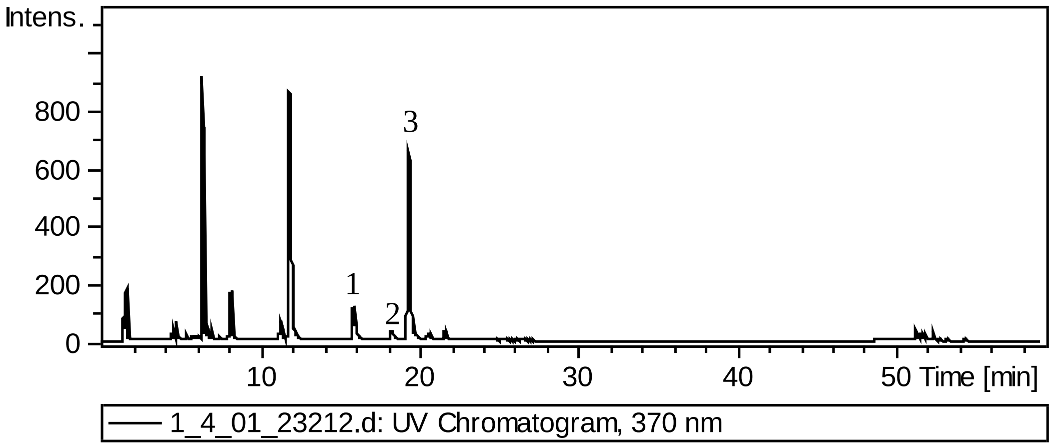


**Supplementary Figure S6 Chromatogram of flavonol glycosides.** Characteristic chromatogram (370 nm) of flavonol glycosides in lettuce grown without polytunnels detected with HPLC-DAD-MS[3]. (1) Quercetin-3-glucuronide; (2) Luteolin-7-glucuronide 2; (3) Quercetin-3-malonylglucoside.

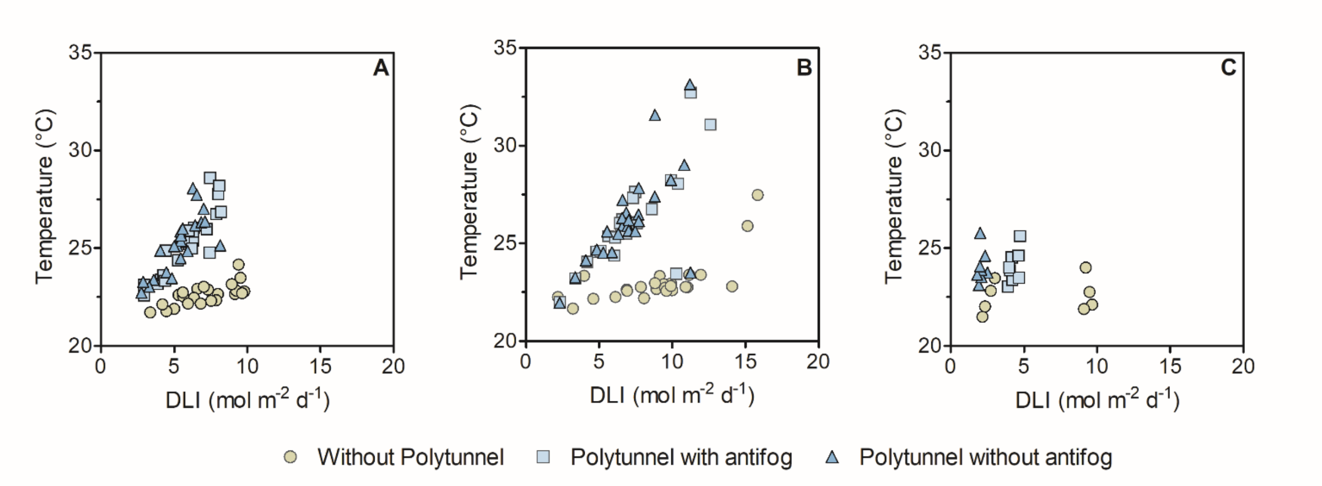


**Supplementary Figure S7 Relationship between light and temperature due to protected cultivation**. Comparisons between cultivation without and with polytunnels. Shown are daily averaged values of the three independent experimental repetitions in (A) April, (B) May, and (C) September.

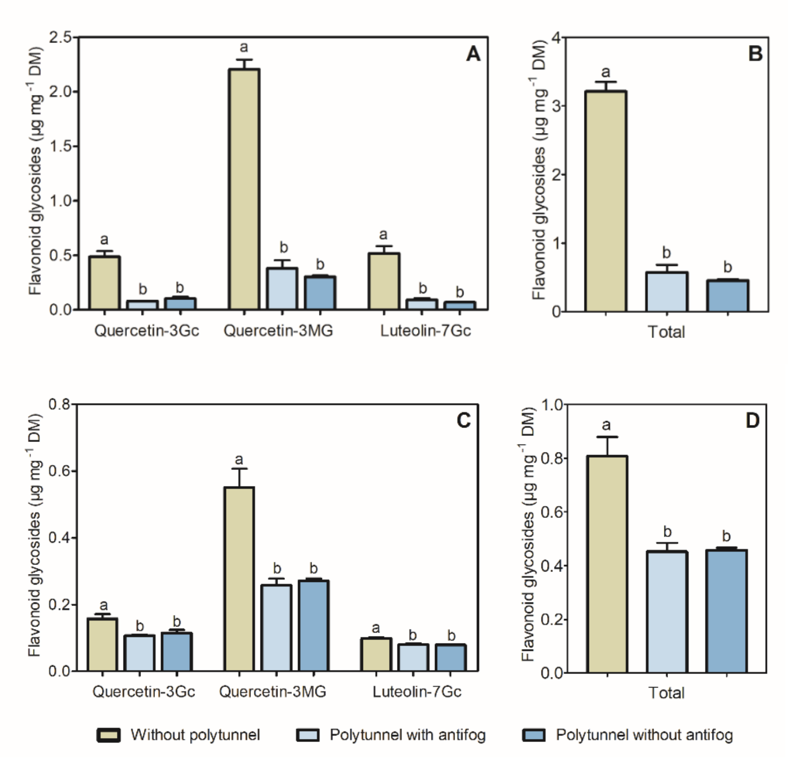


**Supplementary Figure S8 Lower flavonoid content in lettuce grown under polytunnels.** (A,B) The second, and (C,D) third experimental repetition in May and September are shown. (A,C) Individual, and (B,D) total content of flavonoid glycosides (µg $mg^{-1}$ DM) in lettuce grown without and under polytunnels with and without antifogging additives. The data are expressed as mean ± SE

(n = 4). Significant differences ($p \leq 0.05$) of individual and total compounds are indicated by different letters. Gc, glucuronide; MG, malonyl glucoside.

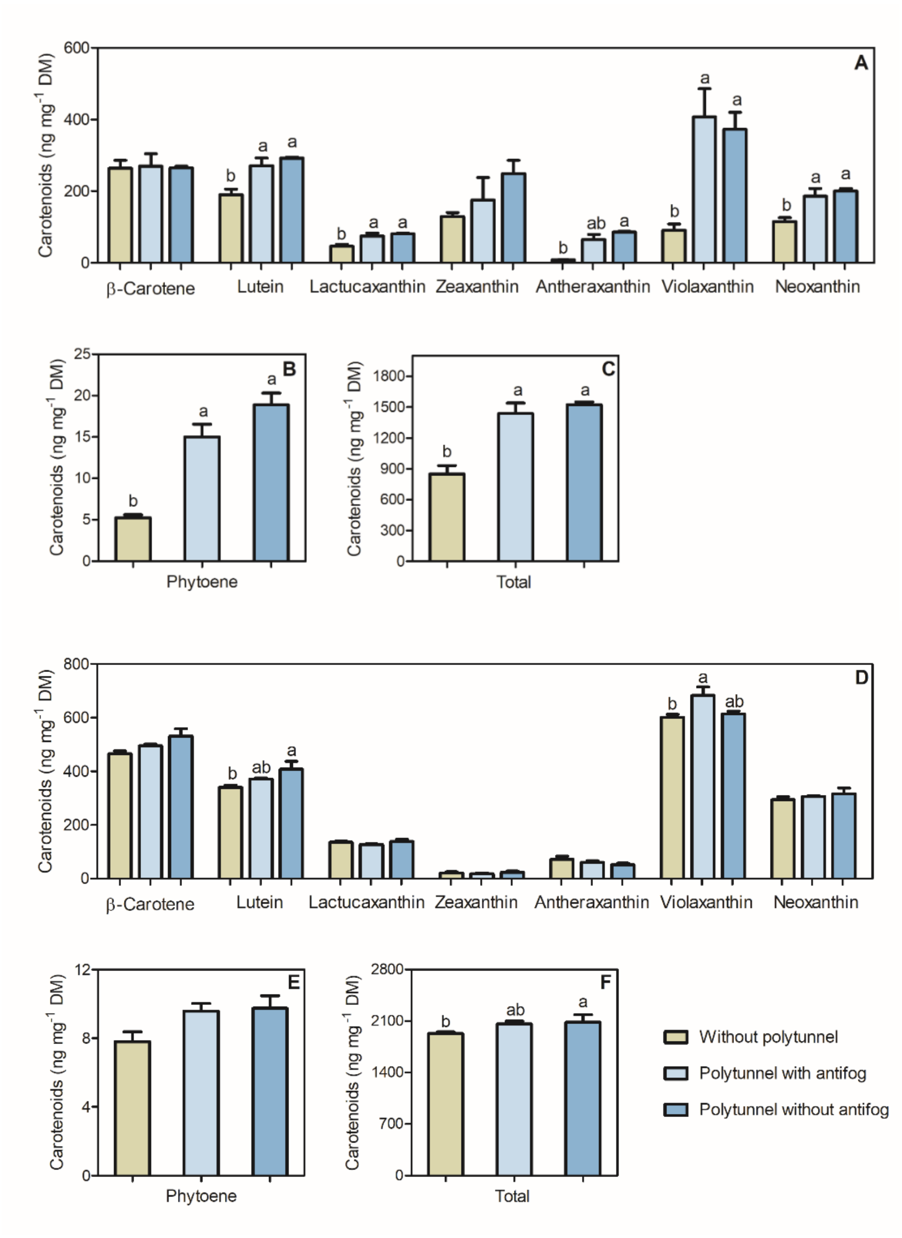


**Supplementary Figure S9 Total carotenoid content affected by polytunnel cultivation.** Carotenoid content (ng $mg^{-1}$ DM) in lettuce grown without or under polytunnels with and without antifogging additives. (A,D) Individual carotenoids β-/ε-branch, (B,E) downstream upper pathway metabolite phytoene, and (C,F) total carotenoids. Second (A-C) and third (D-F) experimental repetition in May and September are shown. The data are expressed as mean ± SE (n = 4). Significant differences ($p \leq 0.05$) of individual and total compounds are indicated by different letters; no letters indicate absence of significance.

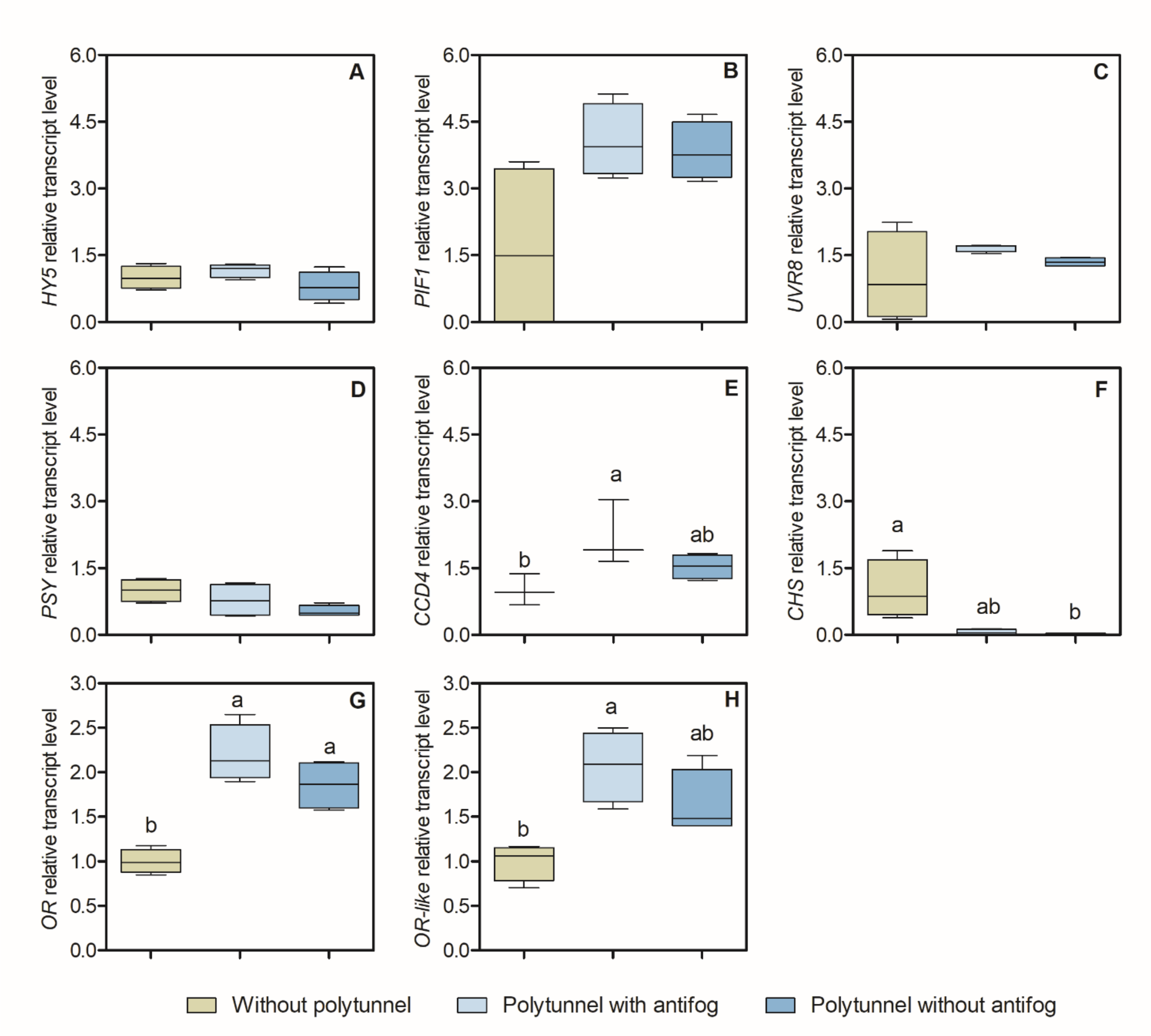


**Supplementary Figure S10 Gene transcripts for key enzymes of the core carotenoid and flavonoid biosynthesis pathways.** Transcript levels of (A) *HY5*, (B) *PIF1*, (C) *UVR8*, (D) *PSY*, (E) *CCD4*, (F) *CHS*, (G) *OR*, and (H) *OR-like* in lettuce grown without or under polytunnels with and without antifogging additives. The second experimental repetition in May is shown. The data are expressed as Box-Whisker-Plots (n = 4), Whiskers show maximal and minimal values. Data was normalized to lettuce grown without polytunnels. Different letters indicate significant differences ($p \leq 0.05$) of transcripts under different cultivation conditions; no letters indicate absence of significance. *HY5*, Elongated hypocotyl5; *PIF1*, Phytochrome interacting factor1; *UVR8*, Ultraviolet resistance locus8; *PSY*, Phytoene synthase; *CCD4*, Carotenoid cleavage dioxygenase4; *CHS*, Chalcone synthase; *OR*, Orange protein; *OR-like*, Orange-like protein.

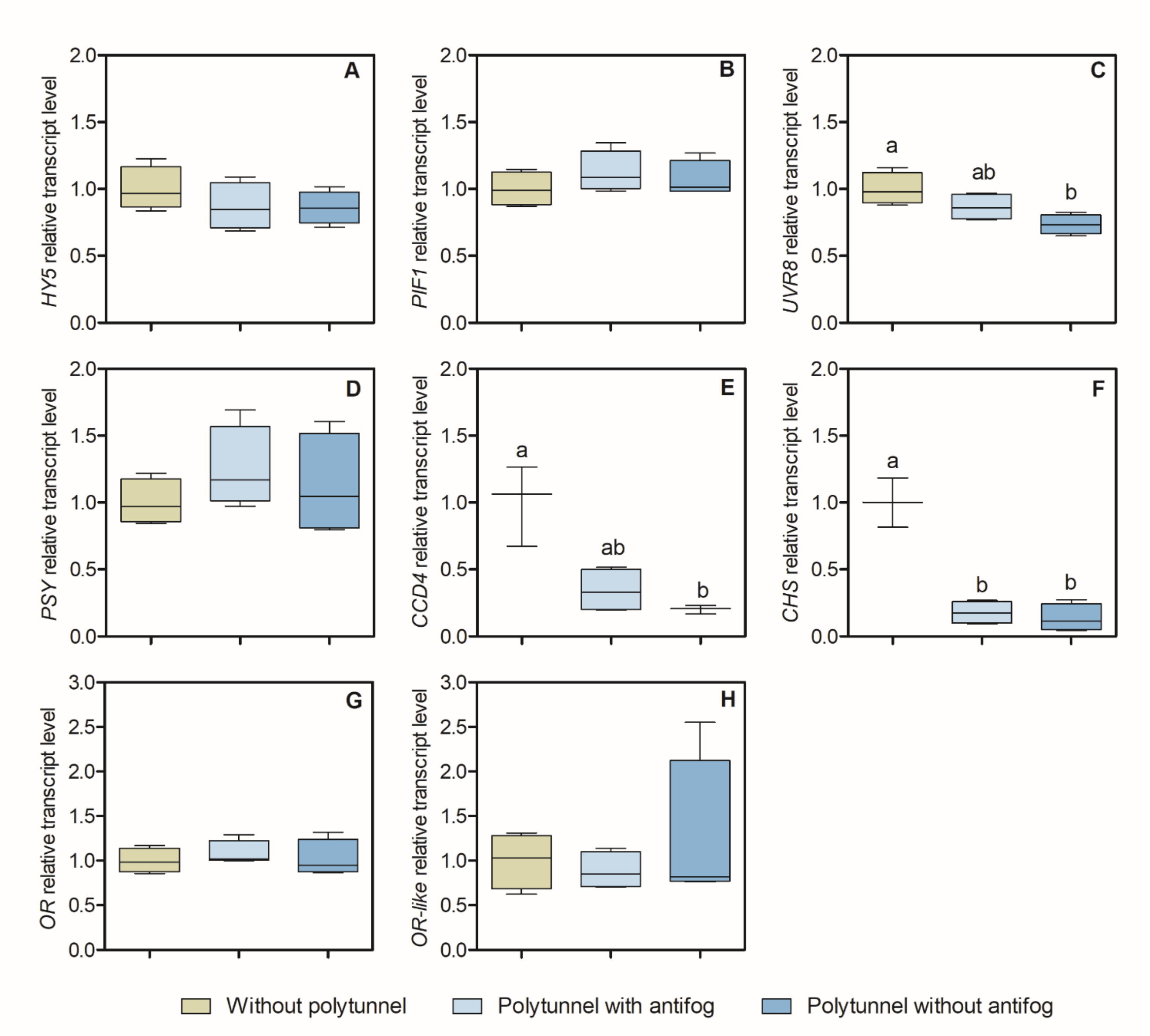


**Supplementary Figure S11 Selected gene transcript for key enzymes of the carotenoid and flavonoid biosynthesis pathways.** Transcript levels of (A) *HY5*, (B) *PIF1*, (C) *UVR8*, (D) *PSY*, (E) *CCD4*, (F) *CHS*, (G) *OR*, and (H) *OR-like* in lettuce grown without or under polytunnels with and without antifogging additives. The third experimental repetition in September is shown. The data are expressed as Box-Whisker-Plots (n = 4), Whiskers show maximal and minimal values. Data was normalized to lettuce grown without polytunnels. Different letters indicate significant differences ($p \leq 0.05$) of transcripts under different cultivation conditions; no letters indicate absence of significance. *HY5*, Elongated hypocotyl5; *PIF1*, Phytochrome interacting factor1; *UVR8*, Ultraviolet resistance locus8; *PSY*, Phytoene synthase; *CCD4*, Carotenoid cleavage dioxygenase4; *CHS*, Chalcone synthase; *OR*, Orange protein; *OR-like*, Orange-like protein.

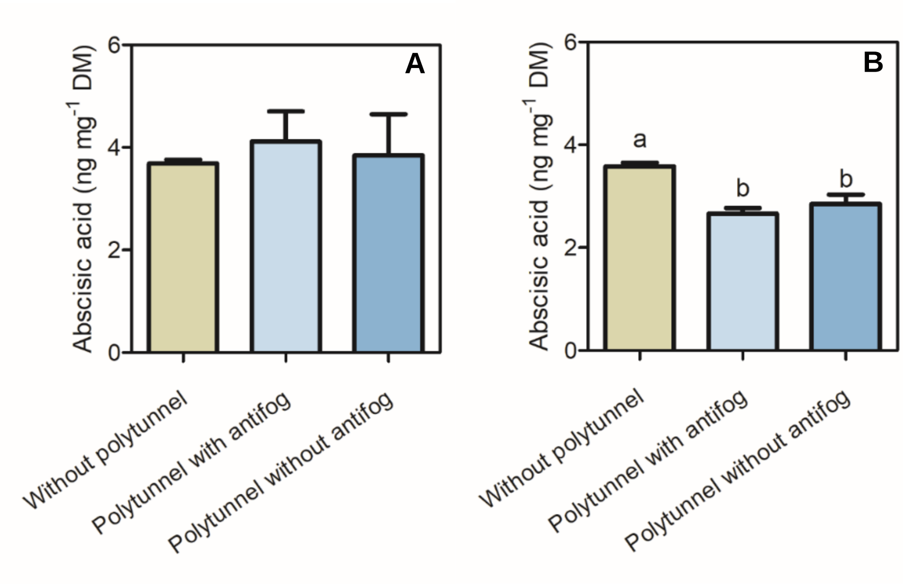


**Supplementary Figure S12 Phytohormone ABA content in lettuce.** Abscisic acid content (ng $mg^{-1}$ DM) in lettuce grown without or under polytunnels with and without antifogging additives. The second (A, May) and third (B, September) experimental repetitions are shown. The data are expressed as mean ± SE (n = 4). Different letters indicate significant differences ($p \leq 0.05$), no letters indicate absence of significance.

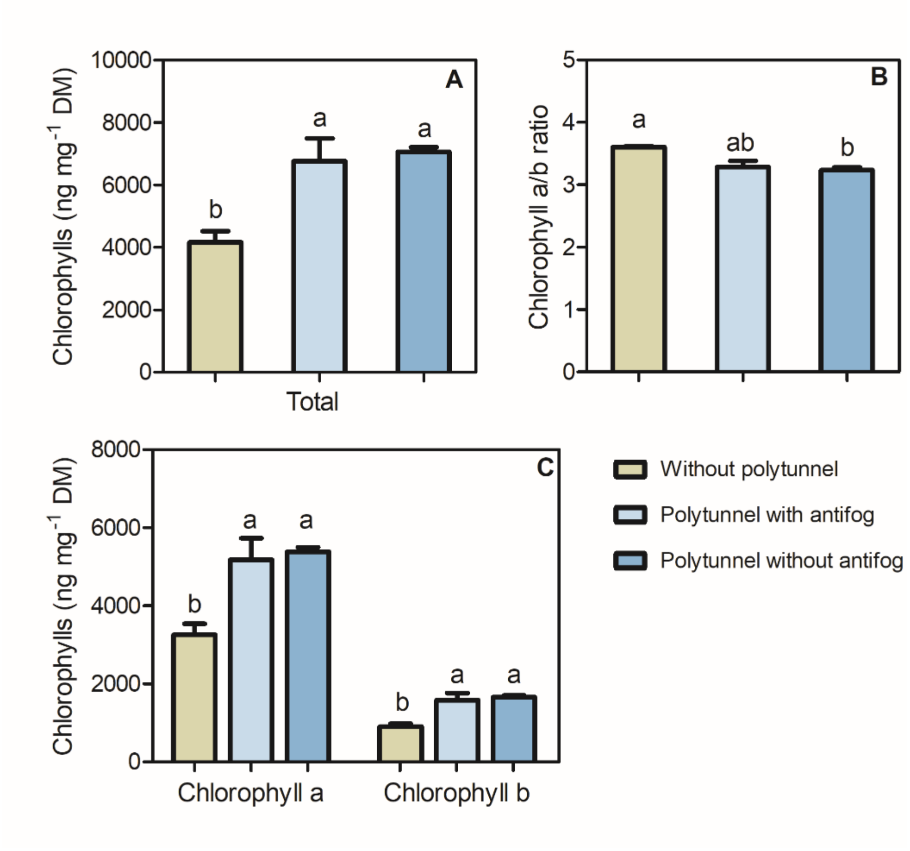


**Supplementary Figure S13 Chlorophyll a and b contents.**
(A) Total, and (C) individual chlorophyll content (ng $mg^{-1}$ DM), and (B) chlorophyll a/b ratio in lettuce grown without and under polytunnels with and without antifogging additives. The first experimental repetition in April is shown. The data are expressed as mean ± SE (n = 4). Significant differences ($p \leq 0.05$) are indicated by different letters.

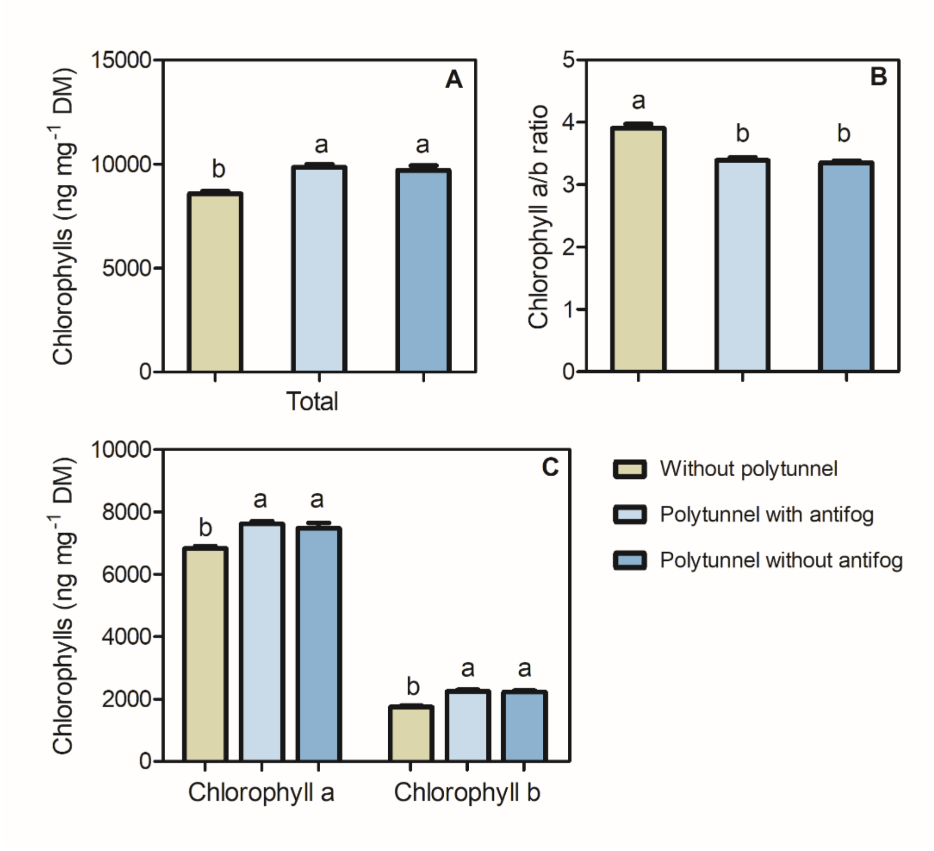


**Supplementary Figure S14 Chlorophyll a and b contents.**
(A) Total, and (C) individual chlorophyll content (ng $mg^{-1}$ DM), and (B) chlorophyll a/b ratio in lettuce grown without and under polytunnels with and without antifogging additives. The second experimental repetition in May is shown. The data are expressed as mean ± SE (n = 4). Significant differences ($p \leq 0.05$) are indicated by different letters.

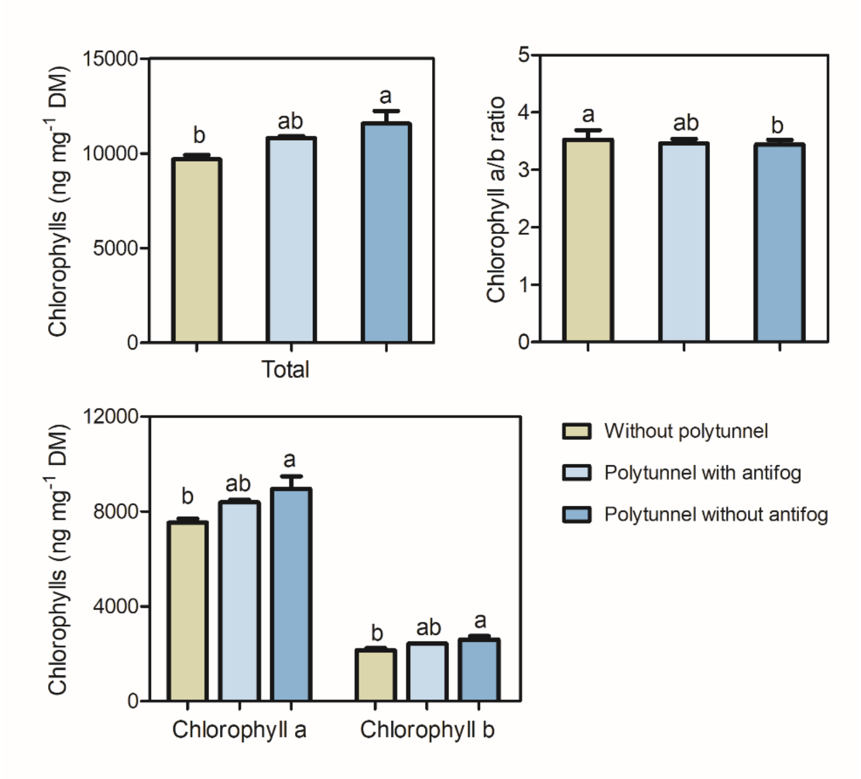


**Supplementary Figure S15 Chlorophyll a and b contents.** (A) Total, and (C) individual chlorophyll content (ng mg$^{-1}$ DM), and (B) chlorophyll a/b ratio in lettuce grown without and under polytunnels with and without antifogging additives. The third experimental repetition in September is shown. The data are expressed as mean ± SE (n = 4). Significant differences ($p \leq 0.05$) are indicated by different letters.

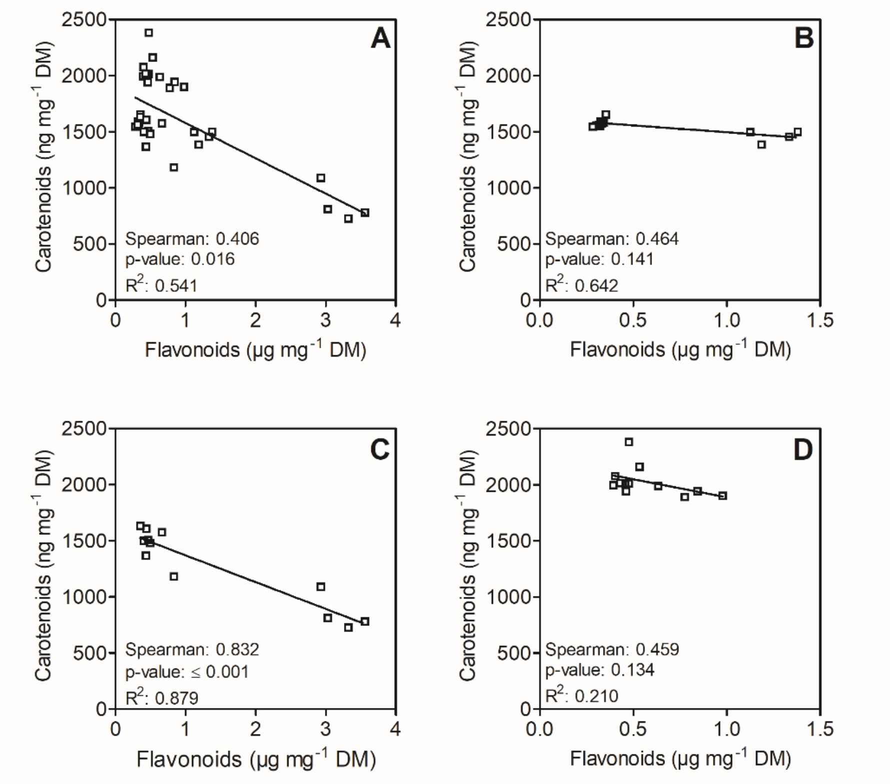


**Supplementary Figure S16 Correlation analysis of carotenoids and flavonoids.** The data represents lettuce grown with protected cultivation, with and without polytunnels (total of n = 12 per repetition). (A) Experimental repetitions 1-3 together, (B) repetition 1 (April), (C) repetition 2 (May), and (D) repetition 3 (September). Because assumption of normal distribution was violated, Spearmans correlation coefficients were determined.

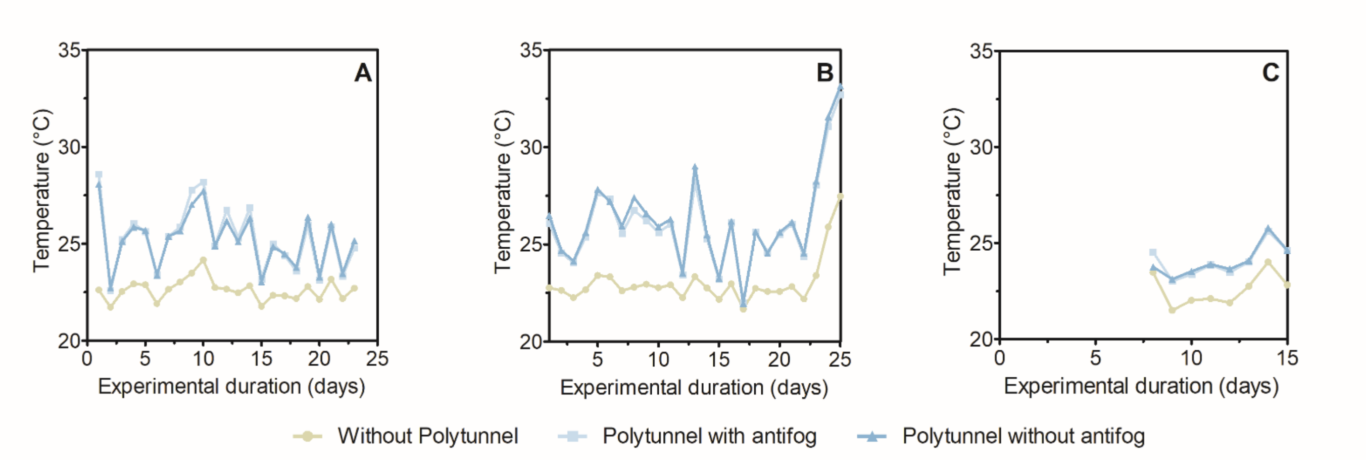


**Supplementary Figure S17 Daily average temperatures**. Daily temperatures averaged in (A) the first (April), (B) second (May), and (C) third experimental repetition (September) for lettuce grown without or under polytunnels with and without antifogging additives. Due to a malfunction of the temperature sensor, the temperature in the third experiment was recorded starting from the middle of the experiment.
